# CD14^+^CXCL10^+^ monocytes are associated with peripheral immune network alterations in systemic juvenile idiopathic arthritis: From multiple centers

**DOI:** 10.1016/j.gendis.2025.101942

**Published:** 2025-11-19

**Authors:** Qiang Luo, Jun Yang, Haiguo Yu, Han Hao, Xinglin Wu, Xiwen Luo, Mingsheng Ma, Xi Yang, Zhiyong Zhang, Yunfei An, Xiaodong Zhao, Hongmei Song, Xuemei Tang

**Affiliations:** a Department of Rheumatology and Immunology, Chongqing Key Laboratory of Child Rare Diseases in Infection and Immunity, National Clinical Research Center for Child Health and Disorders, Ministry of Education Key Laboratory of Child Development and Disorders, Children's Hospital of Chongqing Medical University, Chongqing 400014, China; b Rheumatology and Immunology Department of Shenzhen Children's Hospital, Shenzhen, Guangdong 518100, China; c Department of Rheumatology and Immunology, Nanjing Children's Hospital, Nanjing, Jiangsu 210000, China; d School of Public Health, North China University of Science and Technology, Tangshan, Hebei 063000, China; e Pediatric Department of Peking Union Medical College Hospital, Chinese Academy of Medical Sciences, Beijing 100000, China

**Keywords:** Biomarkers, CD14^+^CXCL10^+^monocyte, Multicenter clinical study, Systemic juvenile idiopathic arthritis, UBE2D1-deficient mice

## Abstract

Systemic juvenile idiopathic arthritis (sJIA) is an autoinflammatory disorder characterized by systemic immune dysregulation, yet reliable biomarkers to predict its unpredictable disease course are lacking. Identifying immune cell subsets and molecular drivers of disease progression is essential for improving prognosis and developing targeted therapies. Here, we performed comprehensive immunophenotypic profiling of PBMCs from sJIA patients across five clinical centers. We identified an unrecognized CD14^+^CXCL10^+^ monocyte subset in sJIA distinguished by a unique transcriptomic signature enriched in immune regulatory genes. Deconvolution analysis with longitudinal follow-up in the Chongqing cohort revealed a previously unrecognized CD14^+^CXCL10^+^ monocyte subset that was markedly expanded during active sJIA and diminished during remission, correlating strongly with disease activity. Flow cytometry confirmed its dynamic changes, and *in vitro* inflammatory stimulation promoted the differentiation of monocytes into the CXCL10 phenotype. To validate these observations *in vivo*, we used Ube2d1 knockout mice, which exhibit impaired CXCL10 induction and attenuated arthritis severity, highlighting the pivotal role of Ube2d1 in driving this inflammatory program. Furthermore, a cross-disease single-cell reference atlas demonstrated that this monocyte subset displayed a distinct expression profile in sJIA compared with other JIA subtypes and inflammation-related diseases. Collectively, our findings indicate that UBE2D1-driven CD14^+^CXCL10^+^ monocytes are central to sJIA pathogenesis and may represent both a biomarker and a therapeutic target for disease monitoring and intervention.

## Introduction

Systemic juvenile idiopathic arthritis (sJIA) is an autoimmune disease in children. Characterized by recurrent, uncontrolled joint inflammation, sJIA causes significant pain, impairs quality of life, and may lead to lasting disability.^1^ Despite the therapeutic advances brought by targeted biologics such as TNF-α inhibitors and conventional immunosuppressants like corticosteroids and methotrexate, 30%–50% of patients fail to achieve optimal clinical responses.^2^ Furthermore, these treatments are associated with substantial adverse effects,^3^ and due to the lack of clear guidelines for safe withdrawal, patients often remain on medications longer than necessary, driven by a high risk of disease flare.^4^ Current treatment strategies are predominantly informed by clinician judgement, with a notable lack of objective biomarkers to reliably assess disease activity.^1^^,^^4^ Consequently, anticipating disease flares, evaluating therapeutic efficacy, and distinguishing true remission from residual subclinical inflammation remain major challenges, particularly in patients with sustained remission.^1^^,^^5^

Clinically applicable biomarkers are critical for guiding treatment decisions and assessing clinical outcomes in sJIA.^6^ Currently, routine biomarkers such as S100A8 and S100A9 are widely used in clinical practice as indicators of active sJIA.^7^, ^8^, ^9^ However, these markers suffer from a lack of specificity, often misclassify a significant number of patients, and have yet to be standardized for broader clinical usage. Other biomarkers, such as IL-18, IL-1β, and IL-6, are known to correlate with disease activity in sJIA.^8^ However, despite their clinical relevance, IL-1β is often undetectable in serum, while the measurement of IL-18 and IL-6 is not routinely performed due to technical challenges in bioassay methodologies.^10^ These limitations hinder the reliable monitoring of disease activity, which may delay timely interventions and impact patient outcomes.^10^ Beyond these conventional inflammatory markers, emerging candidates, such as Galectin-3 and interferon-gamma, have been proposed as potential biomarkers for sJIA.^11^ In addition, in our previous study, we identified UBE2D1 as a promising biomarker: in diagnostic modeling, UBE2D1 achieved high sensitivity (89%) with moderate specificity (78%), and the inclusion of age and sex further improved the sensitivity to 94%.^12^ However, their roles remain under investigation, and there is currently no consensus on their clinical application.

Gene expression profiling and proteomic analysis of peripheral blood mononuclear cells (PBMCs) have been proposed as potential tools for predicting which sJIA patients are likely to progress to more severe forms of the disease.^13^ For instance, a 99-gene RNA signature has been identified in blood samples taken prior to treatment initiation, which can distinguish sJIA patients who are likely to achieve remission on methotrexate from those who will not.^14^ However, the use of large genetic signatures and complex techniques poses challenges for developing standardized, clinically applicable biomarkers.^5^ As a result, there is currently no reliable, validated biomarker in sJIA that can predict disease progression, identify patients at risk of imminent flares, or guide the optimal timing for medication tapering following sustained remission.

In conclusion, integrating sensitive biomarkers may enable personalized therapy and optimize clinical outcomes.^14^ Therefore, this study incorporates single-cell data from five centers to explore the molecular mechanisms and cellular heterogeneity of sJIA more comprehensively, identifying specific cell subpopulations and molecular markers associated with the characteristics of sJIA. Through the follow-up of sJIA patients, we further evaluated the clinical value of these cell subpopulations and molecular markers. In addition, the collagen antibody-induced arthritis (CAIA) mouse model was used for validation, and a cross-disease single-cell atlas was constructed to assess the specificity of sJIA subpopulations and their translational potential. Informed by prior evidence, we also examined UBE2D1 as a hypothesis-driven candidate within this framework to assess its activity linkage and subtype specificity.

## Methods

### Human samples

For single-cell RNA sequencing (scRNA-seq), PBMCs were prospectively collected from 27 patients with sJIA across five centers and 6 healthy controls. In addition, paired PBMC samples were obtained from 2 sJIA patients before and one month after IL-6 inhibitor treatment. Clinical samples were obtained from the following institutions: Children's Hospital of Chongqing Medical University (10 sJIA) (Table S1), Peking Union Medical College Hospital (1 sJIA), Children's Hospital of Nanjing Medical University (3 sJIA), Shenzhen Children's Hospital (4 sJIA), and Cincinnati Children's Hospital Medical Center (9 sJIA) (GSE207633). For cohort definition, we designated the Chongqing scRNA-seq dataset as the internal cohort, while the scRNA-seq datasets obtained from the other four clinical centers were designated as the external cohort. ceAll patients enrolled in the study were diagnosed based on the classification criteria established by the International League of Associations for Rheumatology (ILAR).^15^ For cross-disease comparison, publicly available scRNA-seq datasets of Kawasaki disease (GSE168732) and sepsis (GSE242127) were downloaded from the GEO database, and Blau syndrome (BLAU) were obtained from the Children's Hospital of Chongqing Medical University.

The retrospective observational cohort study was conducted at the Children's Hospital of Chongqing Medical University. The study included hospitalized children newly diagnosed with sJIA (165) and non-sJIA (607) between January 2018 and July 2024 (Table S2). For bulk RNA-seq, PBMCs were collected from 39 patients with other subtypes of JIA, 19 patients with sJIA, and 8 healthy controls.

### Single-cell RNA-seq processing and integration

The gene expression matrices were processed using Seurat and SCTransform. Cells with high mitochondrial or hemoglobin content were excluded. Batch effects were corrected using Harmony, followed by clustering and uniform manifold approximation and projection (UMAP) visualization. Cell identities were assigned based on canonical marker expression.^16^, ^17^, ^18^, ^19^

### Single cell tissue preference assessment

The Ro/e was defined as the ratio of the observed frequency of a meta-cluster in a given tissue to its expected frequency under a uniform distribution. A Ro/e > 1 indicates over-representation of the meta-cluster in that tissue, whereas Ro/e < 1 indicates under-representation.^20^

To assess whether the abundance of specific monocyte subpopulations differed significantly between the disease and control groups, we calculated odds ratios (ORs) with 95% confidence intervals (CIs). For each sample, we derived the number of cells belonging to a given subpopulation (n_cells) and the total number of cells profiled (total). A 2 × 2 contingency table was then constructed, contrasting the counts of the subpopulation versus all other cells in cases and controls. ORs were estimated using Fisher's exact test for robustness in sparse cell counts. For visualization, ORs and CIs were displayed using forest plots on a logarithmic scale, with the vertical reference line corresponding to OR = 1.

### Differentially expressed gene analysis

Differentially expressed gene (DEG) analysis was performed using FindAllMarkers, applying a log fold change cutoff of 0.25 and requiring gene expression in at least 20% of the cells. Kyoto Encyclopedia of Genes and Genomes (KEGG) pathway enrichment analysis was performed on DEGs identified in specific clusters to explore their associated biological functions. Enrichment analysis was conducted using the KEGG database.^21^

### Clustering and annotation of cell sub-populations

To resolve cellular heterogeneity within major immune lineages, we performed single-cell subclustering after SCTransform normalization and dimensionality reduction. Optimal clustering resolutions were determined based on UMAP topology, cluster number, and DEG patterns. Transcriptional programs of the resulting single-cell subclusters were characterized using AUCell-based gene set activity scoring with MSigDB collections (HALLMARK, KEGG, REACTOME). Differentially enriched gene sets were identified via the Wilcoxon rank-sum test with Bonferroni correction and ranked by the log_2_ AUC fold change. Subclusters were annotated by correlating the top-expressed genes with enriched pathways, thereby highlighting dominant regulatory or inflammatory signatures.^22^ The functional enrichment scores were calculated using AUCell-based activity scoring with MSigDB collections (HALLMARK, KEGG, REACTOME), following the approach described by Li et al.^22^ These scores represent aggregate enrichment activity rather than a single pathway. Correlation analysis between enrichment scores and the expression levels of the top 10 highly expressed genes within cell subclusters was performed using Spearman's rank correlation.

### Trajectory analysis

We performed cellular trajectory inference on the integrated scRNA-seq dataset using multiple computational frameworks, including CytoTRACE,^23^ Monocle2,^16^ Monocle3.^24^^,^^25^

### Cell–cell communication and ligand–target regulatory analysis

Intercellular communication networks were inferred using the CellChat and NicheNet R package.^26^^,^^27^

### Immune-related gene set enrichment analysis (irGSEA)

To characterize immune pathway activity at single-cell resolution, we performed immune-related gene set enrichment analysis (irGSEA) using the irGSEA R package (v1.0.1).^28^

### Analysis of candidate genes and signatures of monocyte subsets in the Chongqing cohort

Bayesian deconvolution (BayesPrism v1.1.0) was applied to bulk PBMC RNA-seq data to estimate immune cell fractions.^29^ Clinical relevance was assessed via Spearman correlation with inflammatory markers and the Wilcoxon test for Canakinumab response. Candidate genes were identified by intersecting DEGs from internal, external, and post-treatment sJIA datasets.

### Experimental validation in patient-derived cells and Ube2d1-deficient arthritis models

To validate the transcriptomic findings, a combination of *in vitro* assays and *in vivo* models was employed. Flow cytometry was used to quantify CXCL10 expression in CD14^+^ monocytes from sJIA PBMCs. In THP-1 cells, cytokine (LPS, IL-6, IL-1β, and TNF-α) and chemokine (CCL4) stimulations were performed to assess UBE2D1 and CXCL10 expression dynamics via quantitative polymerase chain reaction (qPCR) and enzyme-linked immunosorbent assay (ELISA). Functional perturbation was further examined through UBE2D1 overexpression and siRNA knockdown, followed by RNA-seq and gene set enrichment analysis.

In parallel, CRISPR/Cas9-engineered Ube2d1 knockout mice were subjected to the collagen antibody-induced arthritis (CAIA) model to evaluate inflammatory phenotypes. Disease assessment included spleen transcriptomic profiling, serum cytokine quantification, and joint ELISAs targeting inflammasome-related components. Structural damage was quantified by high-resolution micro-computed tomography (micro-CT), while multiplex immunofluorescence was employed to visualize immune cell infiltration and CXCL10 expression in joint tissues. In addition, arthritis severity was scored every two days by two independent observers using a standardized 0–4 scale: 0, no redness or swelling; 1, knuckle swelling; 2, mild swelling of the ankle or wrist; 3, pronounced swelling of the entire paw; and 4, joint stiffness or deformity.

All the technical details and full protocols are described in the Supplementary Methods.

## Results

### Single-cell transcriptomics reveals monocyte expansion and immune imbalance in sJIA

In this study, we performed scRNA-seq on 27 patient samples from 5 clinical centers and 6 samples from healthy controls, as well as 4 paired samples from two sJIA patients collected before and one month after IL-6 inhibitor treatment. Additional samples from 16 sJIA patients and 8 controls were used for validation through bulk RNA-sequencing, flow cytometry, and ELISA. Given that sJIA is considered an exclusionary diagnosis, we included clinical data from 168 sJIA patients and 605 patients with other subtypes of JIA, in addition to bulk RNA-seq data from 39 patients with other JIA subtypes, for comparative analysis with sJIA outcomes. Cell type annotation was performed by analyzing the expression levels of canonical gene markers and DEGs**.**

**Figure 1:**
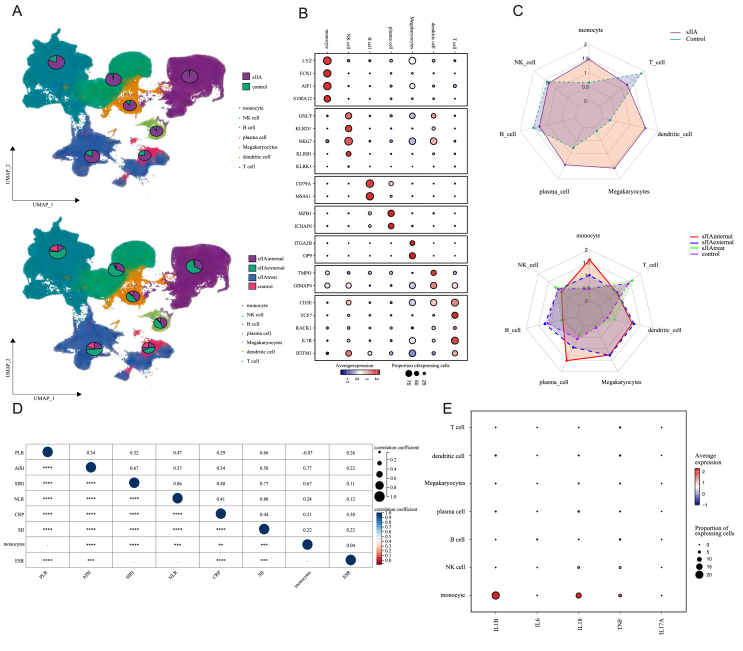
Single-cell atlas of PBMCs from patients with sJIA and controls. **(A)** UMAP visualization of integrated scRNA-seq data, with major immune cell types annotated, including monocyte, NK cell, B cell, plasma cell, megakaryocytes, dendritic cell, and T cell. (Top) sJIA *vs.* control groups. (Bottom) sJIA-internal, sJIA-external, and sJIA-after-treatment *vs.* control groups. **(B)** Dot plot illustrating the expression of canonical marker genes used to annotate major immune cell populations, including monocyte, NK cell, B cell, plasma cell, megakaryocytes, dendritic cell, and T cell. The dot size represents the proportion of cells expressing the gene; the color intensity reflects the average expression level. **(C)** Radar chart showing the distribution of immune cell types in sJIA and controls. (Top) sJIA and control. (Bottom) internal, external, post-treatment sJIA subgroups and controls. **(D)** Dot plot showing the Spearman's correlations of monocyte and clinical features across the Chongqing Clinical Dataset (∗*p* < 0.05, ∗∗*p* < 0.01, ∗∗∗*p* < 0.001, and ∗∗∗∗*p* < 0.0001). Abbreviations: PLR, platelet-to-lymphocyte ratio; AISI, aggregate index of systemic inflammation; SIRI, systemic inflammation response index; NLR, neutrophil-to-lymphocyte ratio; CRP, C-reactive protein; SII, systemic immune-inflammation index; ESR, erythrocyte sedimentation rate. **(E)** Dot plot showing inflammatory cytokine expression across immune cell types in the multicenter sJIA cohort.

After stringent quality filtering and batch effect correction of the scRNA-seq data, 273,671 cells were clustered into 54 clusters with an unsupervised approach, defined by well-established canonical marker genes (Fig. 1A and B and Table S3). These clusters were grouped into the main cellular categories: monocyte, NK cell, B cell, plasma cell, megakaryocytes, dendritic cell, T cell (Fig. 1A).

The cellular proportion results indicate that T cells are significantly greater in the control group and post-treatment sJIA patients compared to those with sJIA. Conversely, the percentage of monocytes was significantly higher in sJIA patients than in healthy controls and was reduced after IL-6 inhibitor treatment (Fig. S1). The Ro/e results demonstrated a strong tissue preference for monocytes in sJIA, as indicated by Ro/e scores greater than 1 in both the internal and external validation sets, whereas in the post-treatment and control groups, the monocyte Ro/e scores dropped below 1 (Fig. 1C). Furthermore, within distinct disease groups, monocytes were significantly higher in sJIA patients than in other subtypes (Fig. S2), and there was a positive correlation between sJIA monocytes and both the inflammation index and CRP (C-reactive protein) (Fig. 1D). Subsequent analysis of genes associated with sJIA, including IL-1β, IL6, IL18, TNF, and IL17A, revealed that IL-1β is primarily expressed in monocytes (Fig. 1E). Clinical cohort data further indicated a positive correlation between sJIA monocytes and IL-1β secretion, a relationship not observed in non-sJIA patients, underscoring the importance of monocytes in sJIA (Fig. S3A). Subsequent KEGG enrichment analysis revealed that pathways associated with sJIA, such as the Toll-like receptor signaling pathway, TNF signaling pathway, and NOD-like receptor (NLR) signaling pathway, were predominantly enriched in monocytes (Fig. S3B; Fig. S4), whereas these pathways were not significantly enriched in other cell types. GO enrichment analysis suggested that monocytes are closely associated with several key biological functions, including the adaptive immune response based on somatic recombination of immune receptors built from the immunoglobulin superfamily domain, the B cell receptor signaling pathway, antigen processing and presentation of exogenous peptide antigens via MHC class II, peptide antigen assembly with the MHC class II protein complex, and MHC class II protein complex assembly (Fig. S3C and Table S4).

### Discovery of a pathogenic CD14^+^CXCL10^+^ monocyte subcluster driving inflammation in sJIA

**Figure 2:**
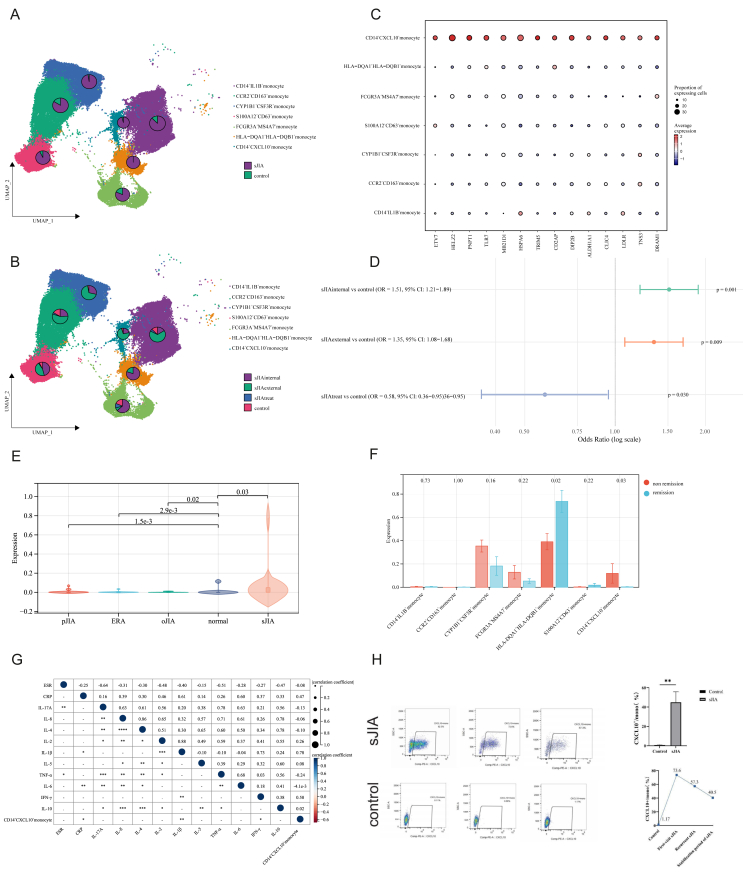
Single-cell landscape of the monocyte components. **(A, B)** UMAP plots showing the distribution of monocyte subpopulations between sJIA and controls (top), and across different disease stages (bottom: sJIA-internal, sJIA-external, sJIA-after-treatment, control). **(C)** Dot plot showing inflammatory cytokine expression across immune cell types in monocyte subtypes. **(D)** Forest plot showing odds ratios (ORs) with 95% confidence intervals (CIs) for the internal, external, and post-treatment sJIA subgroups and controls. **(E)** Violin plot showing CD14^+^CXCL10^+^ monocyte levels across pJIA, ERA, oJIA, controls, and sJIA. Abbreviations: oJIA, oligoarticular juvenile idiopathic arthritis; pJIA, polyarticular juvenile idiopathic arthritis; ERA, enthesitis-related arthritis; **(F)** Differential expression of monocyte subpopulations in sJIA patients with and without remission. **(G)** Dot plot showing the Spearman's correlations of CD14^+^CXCL10^+^ monocytes and clinical features across the Chongqing Clinical Dataset (∗*p* < 0.05, ∗∗*p* < 0.01, ∗∗∗*p* < 0.001, and ∗∗∗∗*p* < 0.0001). **(H)** Flow cytometry analysis of CD14^+^CXCL10^+^ monocytes in the peripheral blood of sJIA patients and controls. Left: Representative flow cytometry plots showing the percentage of CD14^+^CXCL10^+^ monocytes in PBMCs from sJIA patients (top row) and controls (bottom row). Top right: Quantification of the proportions of sJIA patients and controls. Bottom right: Longitudinal analysis of the progression of sJIA disease.

Given that monocytes are highly involved in sJIA pathogenesis, we performed subclustering of monocytes cells to further delineate the pathogenic programs. A total of 7 subtypes were identified (Fig. 2A and B; Fig. S5). The cellular proportion results (Fig. S6) indicate that the CCR2^+^CD163^+^ monocyte subset showed a sharp increase in sJIA but remained at a much lower level in controls, suggesting its involvement in disease-associated inflammation and tissue infiltration. The CYP1B1^+^CSF3R^+^ monocyte subset was notably expanded in sJIA compared to controls. The up-regulation of CSF3R suggests a role in emergency myelopoiesis, while CYP1B1 expression may reflect oxidative stress or metabolic reprogramming under systemic inflammation. The S100A12^+^CD63^+^ monocyte subset exhibited a marked increase in sJIA compared to controls, suggesting an inflammation-prone phenotype. S100A12 is a known damage-associated molecular pattern (DAMP) and proinflammatory marker in systemic autoinflammatory diseases, while CD63 expression indicates vesicle trafficking and degranulation activity. The expansion of this subset supports its potential role in amplifying innate immune responses in sJIA**.**

Interestingly, we identified a novel activated sublining monocyte subcluster (CD14^+^CXCL10^+^) with a distinct transcriptional profile that included high expression of transcription factors (ETV7, HELZ2, and PNPT1), mitochondrial RNA processing genes (PNPT1), stress response genes (HSPA6), cytoskeletal and membrane remodeling regulators (CD2AP, DIP2B, and CLIC4), and innate immune signaling regulators (TRIM5 and TLR7) (Fig. 2C). Functional enrichment analysis of the CD14^+^CXCL10^+^ monocyte subpopulation revealed a complex transcriptional program involving multiple biologically relevant pathways (Fig. S7 and Table S5). Prominently, this population was enriched in immune-related processes, including antigen presentation, activation of innate immune signaling, and pathogen recognition, suggesting a heightened state of immune surveillance and responsiveness (Fig. S7). In addition, CD14^+^CXCL10^+^ monocyte exhibited enhanced activation of oxidative stress responses, ubiquitin–proteasome degradation, and energy metabolism pathways (Fig. S7), indicating a functionally active state capable of responding to inflammatory cues, processing antigens, and undergoing metabolic adaptation.

Subsequently, we calculated the average functional enrichment score for the significant biological processes enriched in CD14^+^CXCL10^+^ monocytes. The correlation of these genes with the top 10 most highly expressed genes within this subset were assessed. This analysis revealed that CXCL10 exhibited the strongest correlation with the functional enrichment scores of these cells, highlighting its pivotal role in the functional attributes of this cell population (Fig. S5). The Ro/e results demonstrated a strong tissue preference for CD14^+^CXCL10^+^ monocytes in sJIA, as indicated by Ro/e scores greater than 1 in both the internal and external validation sets, whereas in the post-treatment and control groups, the CD14^+^CXCL10^+^ monocyte Ro/e scores dropped below 1 (Table S6). We next quantified the relative abundance of CD14^+^CXCL10^+^ monocytes across the disease and control groups by calculating the OR. Compared with the controls, this subset was significantly enriched in both the sJIA-internal cohort (OR = 1.51, 95% CI: 1.21–1.89, *p* = 0.001) and the sJIA-external cohort (OR = 1.35, 95% CI: 1.08–1.68, *p* = 0.009). In contrast, following one month of IL-6 inhibitor treatment, the frequency of CD14^+^CXCL10^+^ monocytes were markedly lower than that in the controls (OR = 0.58, 95% CI: 0.36–0.95, *p* = 0.030) (Fig. 2D). These findings indicate that CD14^+^CXCL10^+^ monocytes are selectively expanded in active sJIA, whereas their abundance decreases upon effective treatment, supporting their pathological relevance and potential value as a treatment-responsive biomarker.

Next, we applied deconvolution analysis to further validate the unique transcriptome and proportions of CD14^+^CXCL10^+^ monocytes in clinical cohorts with bulk RNA-seq information. We found that the number of CD14^+^CXCL10^+^ monocyte was significantly elevated in sJIA patients compared to controls (Fig. 2E). In contrast, the abundance of CD14^+^CXCL10^+^ monocyte was reduced in the other JIA subtypes, including polyarticular juvenile idiopathic arthritis (pJIA), enthesitis-related arthritis (ERA), and oligoarticular JIA (oJIA), compared to the controls (Fig. 2E). And ROC curve analysis revealed an AUC of 0.76 (95% CI: 0.71–0.80), supporting the potential utility of CD14^+^CXCL10^+^ monocytes as a diagnostic biomarker for sJIA (Fig. S8). Following clinical follow-up, sJIA patients were categorized into remission and non-remission groups based on follow-up outcomes. Comparative analysis revealed that CD14^+^CXCL10^+^ monocytes were significantly elevated in the non-remission group compared to the remission group (*p* = 0.03), suggesting their potential association with persistent disease activity (Fig. 2F and Table S7). Finally, in the clinical cohort, we investigated the associations between CD14^+^CXCL10^+^ monocytes and multiple inflammatory and immune-related markers, including the erythrocyte sedimentation rate (ESR), CRP, IL-6, IL-1β, TNF-α, IFN-γ, IL-17As, IL-8, IL-4, IL-2, IL-5, and IL-10 (Fig. 2G). We found that CD14^+^CXCL10^+^ monocytes were positively correlated with CRP, IL-1β, and IFN-γ, supporting their close association with systemic inflammation and interferon-mediated immune responses (Fig. 2G). Finally, flow cytometry analysis revealed that CD14^+^CXCL10^+^ monocytes were significantly increased in sJIA compared to the controls. Notably, their abundance was markedly elevated during the new-onset and relapse phases of sJIA but significantly reduced during the remission phase (Fig. 2H).

### CD14^+^CXCL10^+^ monocytes actively interact with T, NK, and B cells to amplify systemic inflammation

**Figure 3:**
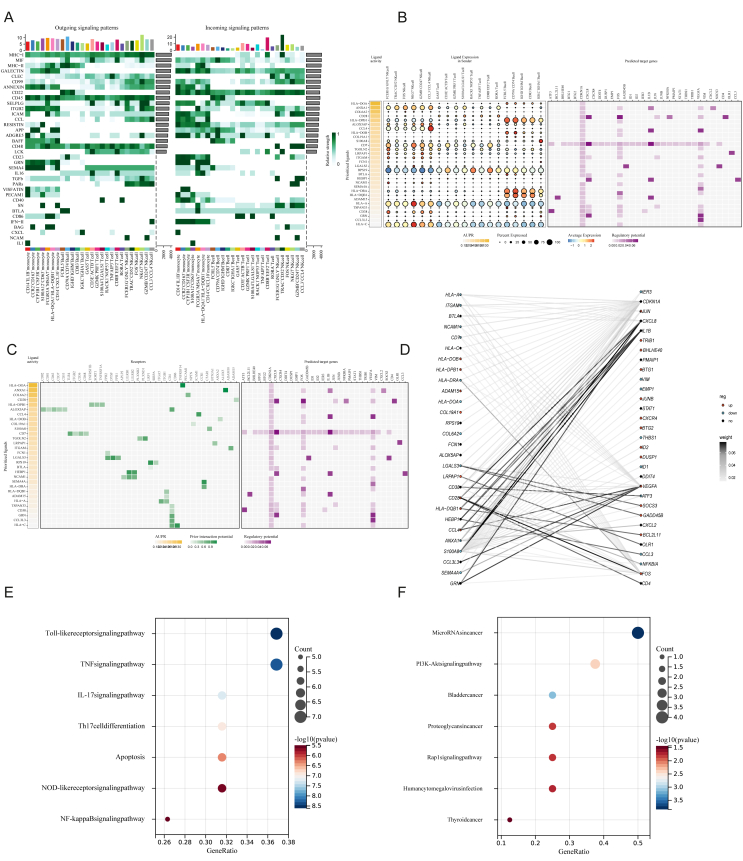
Cell–cell communication patterns among immune cell subtypes in sJIA. **(A)** Heatmap showing the outgoing (left) and incoming (right) signaling pathway activities across different immune cell types. Each row represents a signaling pathway, and each column represents a specific cell population; the color intensity indicates the relative communication strength. **(B, C)** Predicted ligand-receptor-target regulatory network driving inflammatory responses in CD14^+^CXCL10^+^ monocytes. **(D)** Ligand–target regulatory network driving inflammatory activation in CD14^+^CXCL10^+^ monocytes. **(E)** KEGG enrichment analysis of highly expressed genes. **(F)** KEGG enrichment analysis of lowly expressed gene.

To assess the underlying immune–immune crosstalk mechanism in sJIA, we performed intercellular differential ligand–receptor interaction analyses between CD14^+^CXCL10^+^ monocyte subclusters and other immune cell populations in sJIA peripheral blood using CellChat and Nichenet (Fig. S9). The results suggest that CD14^+^CXCL10^+^ monocytes may contribute to shaping the systemic inflammatory milieu of sJIA through multiple immune-regulatory signaling pathways (Fig. 3A). Notably, this subset demonstrated enhanced outgoing activity in the CD45, SEMA4, SN, GRN, BAFF, and APP signaling axes, which are associated with leukocyte activation, cytokine production, and immune cell differentiation. In the incoming signaling landscape, CD14^+^CXCL10^+^ monocytes exhibited a broad yet selective profile of pathway responsiveness. This subset actively receives signals from CD22, CCL, ADGRE5, and GRN, reflecting a diverse range of immunological inputs. These signaling axes are associated with leukocyte activation, chemokine-guided migration, inflammatory regulation, and tissue repair, highlighting the multifaceted immune responsiveness of this subset. For example, CCL signaling is involved in chemokine-guided migration, enabling the positioning of monocytes at inflammatory foci. ADGRE5 (also known as CD97) supports cellular adhesion and tissue localization via interactions with CD55.

NMF-based pattern recognition in outgoing communication assigns CD14^+^CXCL10^+^ monocytes to Pattern 2, a signaling module enriched with pathways such as GALECTIN, ITGB2, RESISTIN, APP, BAFF, GRN, VISFATIN, PECAM1, SN, CD86, BAG, and IL1. These pathways are collectively associated with cell adhesion, inflammatory signaling, immune cell activation, and metabolic–immune regulation, highlighting a complex and multifaceted communication profile of this subset within the inflammatory milieu of sJIA (Fig. S10). In the incoming communication landscape, CD14^+^CXCL10^+^ monocytes were assigned to Pattern 1, characterized by enriched reception of signaling pathways, including MHC-II, ANNEXIN, ICAM, CCL, CD23, GRN, IL16, SEMA4, VISFATIN, PECAM1, CD40, IFN-II, and IL1. These pathways are collectively involved in antigen presentation, cell adhesion, proinflammatory cytokine signaling, and interferon-mediated immune activation, suggesting that CD14^+^CXCL10^+^ monocytes integrate a wide range of inflammatory and immunomodulatory cues from the surrounding cellular environment (Fig. S10).

Thereafter, we identified specific intercellular interactions that were up-regulated in active sJIA samples compared to the controls. The disease-specific interactome revealed that CD14^+^CXCL10^+^ monocytes may engage in immune activation and inflammatory cell recruitment through up-regulated ligand–receptor pairs such as TNFSF13B-TNFRSF13B, RETN-CAP1, ITGB2-ICAM1, and HLA-E-KLRC2 (Fig. S11). Notably, interactions between monocytes and T and NK cells were markedly increased in the active sJIA group, particularly those involving FCRL5^+^ B cells, GZMB^+^CD247^+^ NK cells, and GZMK^+^PRF1^+^ T cells (Fig. S11). To investigate the upstream signaling that drives the activation of CD14^+^CXCL10^+^ monocytes, we applied NicheNet analysis to predict ligand-receptor-target networks. Several ligands, including HLA-DRA, COL6A2, CD28, and CCL4, exhibited high regulatory potential (AUPR) and were predominantly expressed by T and NK cell subsets (Fig. 3B). These ligands were predicted to regulate key inflammatory and stress-responsive genes, such as IL1B, CXCL2, JUNB, NFKBIA, and VEGFA in CD14^+^CXCL10^+^ monocytes (Fig. 3C). To visualize ligand-driven transcriptional responses, we constructed a ligand–target regulatory network based on NicheNet predictions. The network revealed that key ligands, such as CCL4, COL6A2, and CD28, were predicted to regulate multiple inflammation-associated genes, including IL1B, CXCL8, NFKBIA, JUNB, and VEGFA in CD14^+^CXCL10^+^ monocyte (Fig. 3D). To explore the biological pathways downstream of ligand–target interactions, we performed KEGG enrichment analysis on the predicted target genes regulated by the top-ranked ligands. The results revealed that the up-regulated genes were significantly enriched in inflammation- and immune-related pathways, including the TNF signaling pathway, NF-κB signaling pathway, IL-17 signaling pathway, and NLR pathway (Fig. 3E). In contrast, the down-regulated genes were primarily enriched in pathways related to cellular homeostasis and negative regulation of inflammation, such as the PI3K-Akt signaling pathway, suggesting a suppression of anti-inflammatory or regulatory mechanisms in active sJIA (Fig. 3F). Importantly, these ligand–receptor interactions and transcriptional regulatory features were largely absent after treatment, indicating that the CD14^+^CXCL10^+^ monocyte-centered interactome is a disease-specific signature of active sJIA (Fig. S12).

### Trajectory analysis reveals proinflammatory differentiation of monocytes toward a CD14^+^CXCL10^+^ fate in sJIA

**Figure 4:**
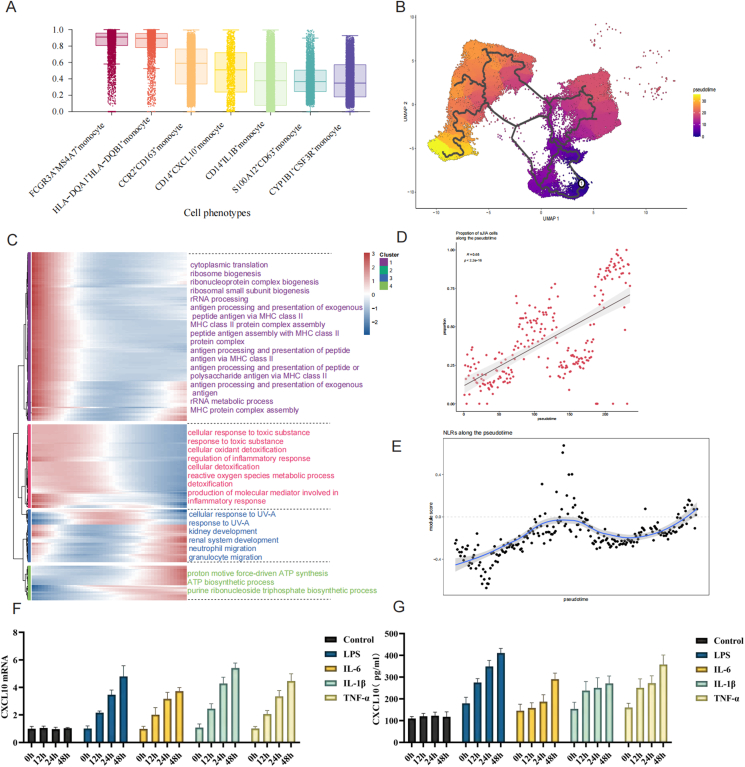
Analysis of monocyte developmental trajectories in sJIA. **(A)** CytoTRACE-based prediction of monocyte subpopulation differentiation potential. **(B)** Pseudotime trajectory analysis of monocyte subpopulations (Monocle3). The color gradient from deep blue through purple, red, and orange to yellow represents the relative pseudotime values assigned to each cell, with deep blue indicating early pseudotime states and yellow corresponding to later stages of pseudotime progression. The black lines denote the principal graph inferred by Monocle3, which delineates the major developmental trajectories across the UMAP embedding. **(C)** Pseudotime-based functional trajectory analysis reveals dynamic enrichment of biological pathways across monocyte subclusters. **(D)** sJIA cell enrichment along the pseudotime trajectory. **(E)** NOD-like receptor signaling (NLR) activation along the pseudotime trajectory. **(F)** Expression of the CXCL10 gene in THP-1 cells under different inflammatory stimuli *in vitro*. **(G)** Expression of the CXCL10 protein in THP-1 cells under different inflammatory stimuli *in vitro*.

Using multiple trajectory inference algorithms, including Monocle2, Monocle3, and CytoTRACE, we identified a robust and consistent monocyte differentiation trajectory. This trajectory originates from stemness-associated FCGR3A^+^HSPA7^+^ monocytes, progresses through intermediate subsets such as HLA-DQA1^+^HLA-DQB1^+^ and CD14^+^CXCL10^+^ monocytes, and terminates in highly inflammatory S100A12^+^CD63^+^ monocytes (Fig. 4A and B).

To investigate the dynamic transcriptional programs along the monocyte pseudotime trajectory, we performed gene module analysis and GO enrichment using Monocle3. Heatmap visualization of enriched biological processes revealed distinct functional states across trajectory-defined modules. Early-stage FCGR3A^+^HSPA7^+^ monocytes (Cluster 1) were enriched in ribosomal biogenesis and antigen presentation, indicating a transcriptionally poised or immune-priming state. Intermediate clusters, including CD14^+^CXCL10^+^ monocyte (Clusters 2, 3), were enriched for pathways related to inflammatory regulation and leukocyte migration, reflecting a transitional proinflammatory phenotype. In contrast, terminal clusters such as S100A12^+^CD63^+^ monocytes (Clusters 4, 5) exhibited marked enrichment in ATP biosynthesis and oxidative phosphorylation, suggesting that enhanced metabolic activity was associated with terminal inflammatory activation (Fig. 4C).

Along the Monocle2-inferred trajectory, two major trajectories (T1 and T2) were identified (Fig. S13). The proportions of CD14^+^CXCL10^+^ monocytes in patients with sJIA increased along the T2 trajectory (Fig. S13). Branch-specific gene expression analysis demonstrated that a group of genes, including NFKBIA, UQCR11, SARAF, GABARAP, IFITM3, FTH1, HLA-B, MARCKS, B2M, SAT1, SRGN, ADGRE5, JAML, TMBIM4, PECAM1, and RSRP1, were progressively up-regulated along the T2 trajectory but showed a declining expression trend along T1 (Fig. S13A). These genes are involved in NF-κB signaling, oxidative stress regulation, MHC class I-mediated antigen presentation, lysosomal activity, and cell adhesion/migration, indicating that T2 represents a transcriptional route toward inflammatory monocyte activation. Additionally, another set of genes (FYB1, RIPOR2, ATP5F1E, VSIR, ATP5MC3, RAB5IF, SOD2, NAMPT, IFITM2, ITM2B, FCGR3B, and GBP5) also showed sustained up-regulation along T2. These genes are associated with mitochondrial ATP synthesis, oxidative stress detoxification, interferon response, and granulocyte-like inflammation, further supporting the notion that T2 reflects a metabolically active and proinflammatory monocyte fate, in contrast to the homeostatic or immune-regulatory phenotype along T1. Consistently, KEGG enrichment analysis revealed significant activation of immune-related pathways, including NLRs (Fig. S13A). In parallel, metabolic pathways such as oxidative phosphorylation, thermogenesis, and nicotinate metabolism were also enriched, supporting a dual program of inflammatory activation and metabolic reprogramming in T2-directed monocytes (Fig. S13A).

To explore the clinical relevance of the pseudotime trajectory, we examined the distribution of sJIA-derived monocytes across the trajectory. The proportion of sJIA cells was found to increase significantly along pseudotime (R = 0.65, *p* < 2.2e−16), indicating that disease-associated monocytes preferentially occupy later stages of the trajectory. Moreover, gene module scoring revealed that NLR pathway activity also increased toward the end of the trajectory, consistent with the inflammatory reprogramming observed in the T2 branch (Fig. 4D and E).

Based on the pseudotime trajectory, CD14^+^CXCL10^+^ monocytes were enriched at the terminal stage of the T2 branch, characterized by inflammation. To validate the functional drivers of this phenotype, we stimulated monocytes with inflammatory mediators *in vitro* (Fig, 4F, G). The results showed a significant time-dependent induction of CXCL10 expression at both the mRNA and protein levels, particularly under LPS and IL-1β stimulation, confirming that CXCL10 is not constitutively expressed but induced by inflammatory cues. These findings support that the CXCL10^+^ monocytes observed at the terminal end of the pseudotime trajectory likely represent inflammation-induced activated monocyte states, consistent with their enrichment in sJIA and high expression of NLR and cytokine signaling pathways.

### UBE2D1 defines the inflammatory transcriptomic program of CD14^+^CXCL10^+^ monocytes

**Figure 5:**
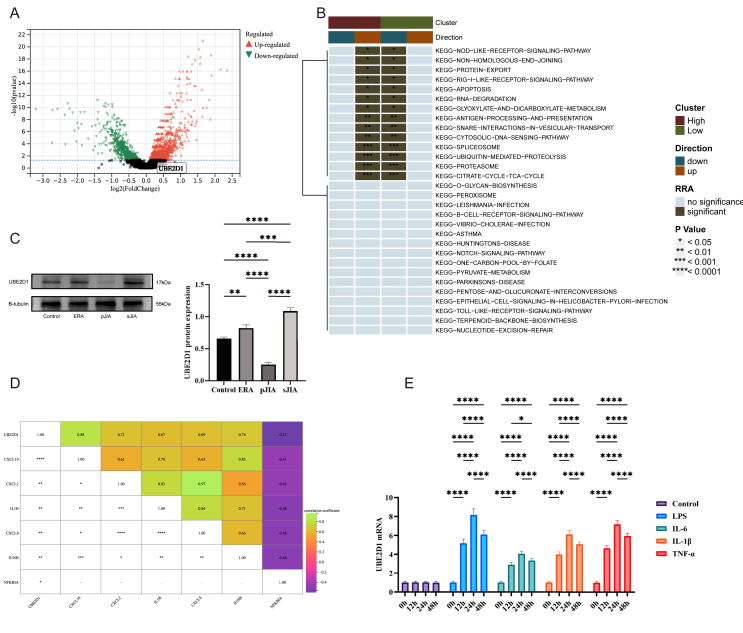
Molecular characteristics of CD14^+^CXCL10^+^ monocytes. **(A)** Differential expression (DEG) analysis in post-treatment sJIA and controls. **(B)** Pathway alterations associated with elevated UBE2D1 expression in monocytes, as determined using irGSEA. Monocytes were stratified into “high” and “low” UBE2D1 clusters according to the mean expression value of UBE2D1, with cells above the mean classified as “high” and those below classified as “low”. **(C)** UBE2D1 protein expression in enthesitis-related arthritis (ERA), polyarticular JIA (pJIA), and systemic JIA (sJIA) and controls (∗*p* < 0.05, ∗∗*p* < 0.01, ∗∗∗*p* < 0.001, and ∗∗∗∗*p* < 0.0001). **(D)** Dot plot showing the Spearman's correlations of UBE2D1 and feature genes (∗*p* < 0.05, ∗∗*p* < 0.01, ∗∗∗*p* < 0.001, and ∗∗∗∗*p* < 0.0001). **(E)** Proinflammatory stimuli induce UBE2D1 expression in monocytes.

To further define the molecular characteristics of CD14^+^ CXCL10^+^ monocytes, we performed differential expression analyses in two independent cohorts (external and internal), each contrasting sJIA with healthy controls. The overlap of 1785 concordant DEGs (Fig. S14) highlights a robust and reproducible sJIA-associated transcriptional signature. In the treatment cohort, many of these disease-associated genes, such as UBE2D1, were no longer differentially expressed compared with those in the controls (Fig. 5A), indicating that their dysregulation is closely linked to active disease and tends to normalize upon clinical remission. KEGG enrichment analysis of DEGs from both internal and external datasets revealed significant enrichment in NLR signaling, Toll-like receptor, and NF-κB signaling pathways, all of which are central to innate immune activation in sJIA. However, these inflammatory signaling pathways were no longer enriched in post-treatment samples, indicating a resolution of inflammatory transcriptional programs following therapy (Fig. S15).

Next, hallmark enrichment analysis based on irGSEA was performed for UBE2D1, revealing several statistically significant pathways that highlight critical biological mechanisms. For instance, in NLRs pathway, UBE2D1 may regulate inflammatory responses by interacting with cytoplasmic pattern recognition receptors (Fig. 5B). In the RIG-I-like receptor signaling pathway, UBE2D1 could affect antiviral immunity by modulating ubiquitination processes involved in RNA virus recognition and downstream interferon signaling. Furthermore, the role of UBE2D1 in the apoptosis pathway suggests its impact on programmed cell death, which is essential for controlling immune responses and maintaining tissue homeostasis. Together, these pathways highlight that CD14^+^CXCL10^+^ monocytes with high UBE2D1 expression exhibit significant upregulation of NLRs, antigen processing and presentation, apoptosis, and RNA degradation pathways (Fig. 5B), suggesting that UBE2D1^high^ genes in CD14^+^CXCL10^+^ monocytes are transcriptionally enriched for innate immune activation, antigen presentation, and stress response programs.

Western blot (WB) analysis was further performed to examine UBE2D1 protein expression across different JIA subtypes (Fig. 5C). The results revealed clear heterogeneity among the subtypes: UBE2D1 expression was markedly elevated in the sJIA group, whereas other JIA subtypes displayed much lower levels. Quantitative analysis confirmed that the up-regulation of UBE2D1 in sJIA was statistically significant compared with that in the non-sJIA subtypes (*p* < 0.0001). These findings demonstrate that UBE2D1 protein expression varies substantially across JIA subtypes and further highlight its specificity for sJIA. To investigate UBE2D1-associated molecular programs, we performed bulk RNA sequencing on THP-1 cells with high and low UBE2D1 expression, followed by differential expression and pathway enrichment analyses. GSEA revealed that UBE2D1-high cells exhibited significant enrichment in multiple immune and inflammatory pathways, including the NLR pathway, cytokine–cytokine receptor interaction, interleukin signaling (Fig. S16A–S16C). Notably, the NLR pathway was one of the top enriched signatures, and was consistently up-regulated in the UBE2D1-high group across the DEG-based and expression-ranked GSEA analyses. These pathways are known to drive proinflammatory responses, suggesting that UBE2D1 up-regulation amplifies innate immune activation, potentially via the enhancement of NLR inflammasome signaling and cytokine-driven inflammation.

Subsequently, based on our prior findings from cell–cell communication and pseudotime trajectory analyses, we evaluated the correlation between UBE2D1 expression and key inflammatory genes, including CXCL10, CXCL2, IL1B, CXCL8, JUNB, and NFKBIA. The results showed that UBE2D1 expression was positively correlated with CXCL10, CXCL2, IL1B, CXCL8, and JUNB, whereas it exhibited a negative correlation with NFKBIA (Fig. 5D). In addition, UBE2D1 expression was also up-regulated following CCL4 intervention, suggesting that UBE2D1 may be a downstream target of CCL4-mediated signaling, further implying that UBE2D1 may participate in the transcriptional regulation of CCL4-induced inflammatory responses (Fig. S17). Finally, *in vitro* experiments showed that stimulation of THP-1 monocytes with LPS, IL-1β, and TNF-α led to a time-dependent up-regulation of UBE2D1 expression, further validating its responsiveness to proinflammatory stimuli (Fig. 5E).

**Figure 6:**
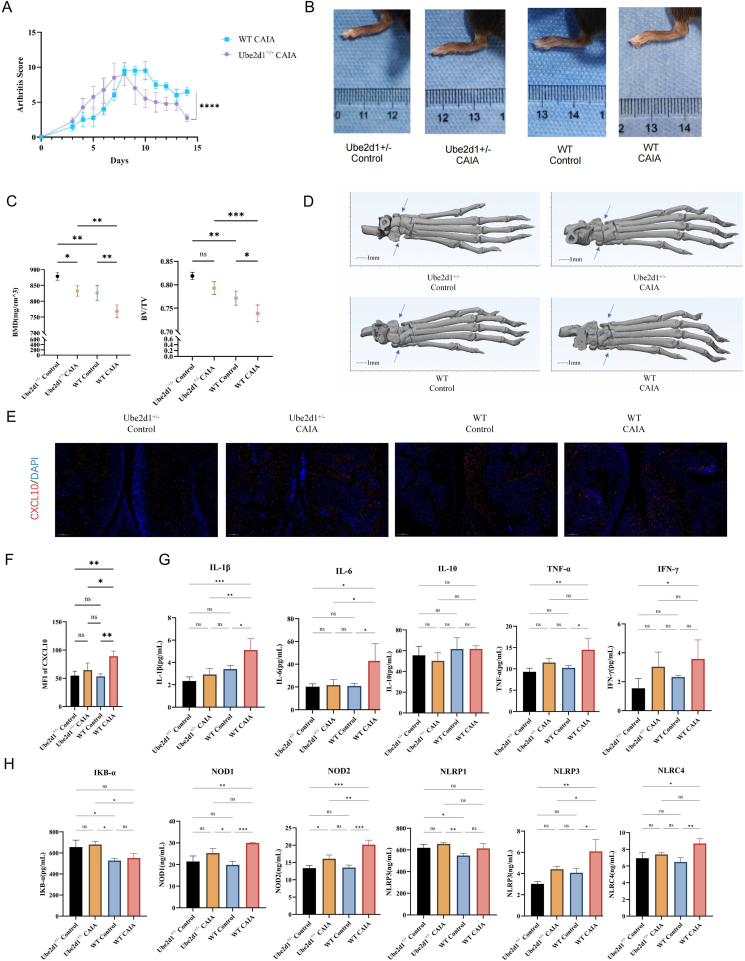
Ube2d1^+^/^−^ mice in the collagen antibody-induced arthritis (CAIA) model. **(A)** Time-course plot showing arthritis scores over 14 days: Ube2d1^+^/^−^ CAIA mice and WT CAIA mice (∗∗∗∗*p* < 0.0001). **(B)** Representative images of hind paw swelling in different groups: Ube2d1^+^/^−^ control, Ube2d1^+^/^−^ CAIA, WT control, and WT CAIA. **(C)** Quantitative micro-CT analysis of bone parameters in Ube2d1^+^/^−^ and WT mice with or without CAIA induction. Bone mineral density (BMD, left) and bone volume/tissue volume ratio (BV/TV, right) were measured in the hind paw joints (∗*p* < 0.05, ∗∗*p* < 0.01, ∗∗∗*p* < 0.001, and ∗∗∗∗*p* < 0.0001). **(D)** Representative micro-CT 3D reconstructions of ankle joints: Ube2d1^+^/^−^ control, Ube2d1^+^/^−^ CAIA, WT control, and WT CAIA. **(E)** Representative multiplex immunofluorescence staining images of joint tissues from four experimental groups: Ube2d1^+^/^−^ control, Ube2d1^+^/^−^ CAIA, WT control, and WT CAIA. **(F)** Quantification of CXCL10 expression in joint tissues (∗*p* < 0.05, ∗∗*p* < 0.01, ∗∗∗*p* < 0.001, and ∗∗∗∗*p* < 0.0001). **(G)** The expression levels of cytokines related to sJIA in Ube2d1^+^/^−^ control, Ube2d1^+^/^−^ CAIA, WT control, and WT CAIA (∗*p* < 0.05, ∗∗*p* < 0.01, ∗∗∗*p* < 0.001, and ∗∗∗∗*p* < 0.0001). **(H)** The expression levels of NOD-like receptor signaling (NLR) pathway proteins related to sJIA in Ube2d1^+^/^−^ control, Ube2d1^+^/^−^ CAIA, WT control, and WT CAIA (∗*p* < 0.05, ∗∗*p* < 0.01, ∗∗∗*p* < 0.001, and ∗∗∗∗*p* < 0.0001).

In addition, we employed Ube2d1 knockout mice in a CAIA model to investigate its functional role *in vivo* (Fig. 6). Compared to WT-CAIA mice, Ube2d1^+^/^−^ mice exhibited significantly lower arthritis scores, indicating attenuated joint inflammation (Fig. 6A and B). Similarly, micro-CT evaluation demonstrated diminished bone erosion and preservation of the bone structure in Ube2d1^+/−^ CAIA mice, with higher bone mineral density (BMD) and bone volume/tissue volume (BV/TV) ratios than WT-CAIA mice (Fig. 6C and D). Immunohistochemistry further showed that the number of CXCL10 was significantly elevated in WT CAIA joints but substantially reduced in Ube2d1^+^/^−^ mice, suggesting impaired recruitment or activation of inflammatory macrophages (Fig. 6E and F). In contrast, Ube2d1^+^/^−^ control mice showed no pathological changes. Multiplex cytokine analysis revealed markedly lower levels of proinflammatory mediators, including IL-1β and IL-6, in the serum of Ube2d1^+^/^−^ mice than in that of WT-CAIA mice (Fig. 6G). Transcriptomic profiling of the spleens from these mice revealed a strong positive correlation between Ube2d1^+^/^−^ and Cxcl10 expression, and pathway enrichment analysis showed that NLR signaling activity was significantly diminished in the Ube2d1^+^/^−^ group (Fig. S18A and S18B). These transcriptomic trends were consistent with the ELISA findings, which confirmed decreased levels of key NLR pathway proteins in Ube2d1-deficient animals (Fig. 6H).

These findings support the hypothesis that UBE2D1 may act as a regulatory component contributing to the transcriptional activation of these genes, potentially serving as a mediator or amplifier of inflammatory signaling.

### CD14^+^CXCL10^+^ monocytes and UBE2D1 expression are selectively enriched in sJIA compared with related diseases

To investigate the disease specificity and diagnostic value of the identified CD14^+^CXCL10^+^ monocyte subset, we compared its distribution across sJIA and other clinically relevant inflammatory diseases that often present with overlapping symptoms, including BLAU, Kawasaki disease (KD), and sepsis (Fig. S19). UMAP visualization of CD14^+^CXCL10^+^ monocytes across BLAU, KD, sJIA, and sepsis revealed a marked increase in their abundance in sJIA, suggesting potential disease-specific accumulation (Fig. S20A–S20D). Ro/e analysis showed that CD14^+^CXCL10^+^ monocytes were most enriched in the sJIA samples, with lower enrichment in BLAU and minimal representation in IVIG and sepsis (Fig. S20E). Finally, we assessed the expression of UBE2D1 across BLAU, KD, sJIA, and sepsis. UBE2D1 expression was markedly elevated in sJIA, clearly distinguishing it from the other disease groups (Fig. S20F). These findings suggest that UBE2D1 is selectively up-regulated in sJIA and may play a role in mediating disease-specific immune responses.

## Discussion

Autoimmune diseases are characterized by a complex pathophysiology driven by the interactions of multiple cellular components and distinct transcriptomic signatures, which collectively influence disease progression and clinical outcomes.^30^ In this context, the integration of advanced techniques such as scRNA-seq and bluk-seq, alongside animal model validation, provides a multidimensional approach to comprehensively dissect the underlying cellular ecosystem in sJIA. Furthermore, the multi-center design of the study enhances the generalizability of the findings, offering more robust and reliable insights. We identified a novel subset of activated monocytes (CD14^+^CXCL10^+^), which plays a key role in regulating the proinflammatory response and mediating signal transmission to neighboring cells. This subset is closely associated with the progression of sJIA and is significantly correlated with patient prognosis.

Unlike other diseases, in sJIA, monocytes and macrophages in children exhibit persistent activation.^31^ However, the precise role of these cells in systemic hyperinflammation remains unclear. In this study, we identified a novel subset of activated monocytes (CD14^+^CXCL10^+^). CXCL10^+^ was found to be involved in autoimmune neuroinflammation and associated monocytes to promote inflammatory responses and induce cytokine storms.^32^^,^^33^ Integrated scRNA-seq data and functional validation revealed the potential multifaceted functions of this subset of monocytes, including metabolism, organelles and cytoplasmic processes, protein modification and degradation, and immune regulation, suggesting the central role in sJIA in persistent inflammation. In addition, the unique expression of ETV7, HELZ2, PNPT1, TLR7, MB21D1 (cGAS), HSPA6, TRIM5, CD2AP, DIP2B, ALDH1A1, CLIC4, LDLR, TNS3, and DRAM1 in CD14+CXCL10+ monocytes suggests that this monocyte subset may increase susceptibility to chronic inflammatory diseases during the chronic stage of the disease by promoting inflammatory responses and reprogramming cellular metabolism. This unique genetic signature implies that this monocyte subset may enhance self-nucleic acid sensing and the production of inflammatory cytokines, similar to the type I interferon production mechanisms observed in systemic lupus erythematosus, potentially laying the groundwork for a cytokine storm, ultimately exacerbating autoimmunity and hindering resolution.^34^

Currently, the primary goal in managing sJIA is to achieve disease remission or maintain a low disease activity state.^35^ However, there is still a lack of precise biomarkers to predict whether the disease has truly entered remission or is in a subclinical state.^36^ Monocytes, which play a key role in regulating immune responses in both diseased and healthy individuals, have always been attractive candidates for biomarkers.^37^ However, no biomarkers specifically targeting monocytes have been established.^37^ One possible explanation is that in sJIA, monocytes often exhibit a mixed pattern of classical and alternative activation, which could indeed affect their utility as biomarkers.^38^ Validated by the cohort from Chongqing Medical University, our analysis indicated that the CD14^+^CXCL10^+^ monocyte subgroup, which possesses a unique transcriptome, could potentially predict the onset of sJIA, effectively differentiate between different disease stages of sJIA, and is closely related to the prognosis of sJIA patients. Furthermore, our study integrates multiple lines of evidence to demonstrate that CD14^+^CXCL10^+^ monocytes represent an inflammation-inducible proinflammatory subset. *In vitro* stimulation of THP-1 monocytes with LPS and various inflammatory cytokines led to a progressive, time-dependent up-regulation of CXCL10 expression, highlighting the sensitivity of this phenotype to inflammatory cues. Previous reports suggest that LPS promotes NLRP3, NOD, and NF-κB activation and enhances NLRs, thereby facilitating CXCL10 up-regulation, particularly under acute inflammatory conditions.^39^, ^40^, ^41^ This regulatory axis was further supported by our single-cell RNA sequencing analysis. Pseudotime trajectory reconstruction revealed sustained activation of the NLR signaling pathway during the differentiation of monocytes into the CD14^+^CXCL10^+^ phenotype, suggesting that this subset acquires its inflammatory features through a transcriptionally dynamic process. Complementary cell–cell communication analysis indicated that T cells and NK cells may serve as upstream inducers by secreting CCL4, which in turn activates NLR signaling and downstream IL1B expression in CD14^+^CXCL10^+^ monocytes, establishing an intercellular inflammatory amplification loop. These mechanistic findings were corroborated by clinical data: flow cytometric profiling showed that the CD14^+^CXCL10^+^ monocyte population was markedly elevated during the active and relapsed phases of sJIA, diminished during remission, and remained persistently high in treatment-refractory patients, implicating this subset not only in disease onset but also in sustained inflammation and recurrence. Taken together, our data suggest that CD14^+^CXCL10^+^ monocytes are an constitute proinflammatory subset dynamically shaped by inflammatory signals, and may serve as critical mediators of the pathogenesis, persistence, and therapeutic resistance of sJIA.

In addition, further exploration of the regulatory landscape of CD14^+^CXCL10^+^ monocytes identified UBE2D1 as a functionally relevant upstream mediator. *In vitro* assays demonstrated that the expression of UBE2D1 gradually increased with LPS over time and was positively correlated with the expression of CXCL10. Moreover, stimulation of THP-1 monocytes with recombinant CCL4 significantly up-regulated UBE2D1 expression, suggesting that UBE2D1 may act as a downstream effector within the CCL4-driven activation cascade in inflammatory monocytes. To further elucidate its functional role in inflammatory signaling, we performed GSEA based on RNA-seq data stratified by UBE2D1 expression levels. GSEA revealed significant enrichment of the NLR pathway in UBE2D1-high cells, providing transcriptomic evidence supporting a mechanistic link between UBE2D1 activity and NLR-mediated inflammation. Collectively, these findings suggest that UBE2D1 not only is a passive responder to inflammatory stimuli but also functions as a key amplifier of inflammatory programs in CD14^+^CXCL10^+^ monocytes, potentially by modulating NLR signaling and ubiquitination-dependent immune pathways. This regulatory association was further validated in UBE2D1 knockout mice.^42^^,^^43^

Limitations of this study include the relatively limited sample size in certain patient subgroups, which may constrain the statistical power to fully capture transcriptomic heterogeneity and its association with clinical features such as treatment resistance or disease recurrence. Additionally, although our multi-level validation, including scRNA-seq, *in vitro* stimulation, and UBE2D1 knockout mouse models, provides strong mechanistic support, the functional role of CD14^+^CXCL10^+^ monocytes in human sJIA pathogenesis warrants further confirmation in prospective longitudinal cohorts and additional tissue-level validation. Future integration of bulk and single-cell transcriptomic deconvolution with spatial immune profiling across multiple disease contexts may offer a more comprehensive view of this inflammatory axis.

In summary, our integrative single-cell and functional study provides a valuable resource for understanding sJIA pathogenesis, highlighting a novel inflammation-inducible CD14^+^CXCL10^+^ monocyte subset as a potential driver of disease activity and recurrence. We identified UBE2D1 as a key upstream modulator of this subset, which is mechanistically linked to NLR signaling, and validated it across multiple platforms, including UBE2D1-deficient mice. This study not only advances our understanding of peripheral immune activation in sJIA but also lays the groundwork for future translational research aimed at biomarker-guided monitoring and precision-targeted therapy in autoinflammatory diseases.

## CRediT authorship contribution statement

**Qiang Luo:** Writing – review & editing, Writing – original draft, Methodology, Investigation. **Jun Yang:** Writing – review & editing, Writing – original draft, Validation. **Haiguo Yu:** Visualization, Methodology, Investigation. **Han Hao:** Validation, Investigation, Data curation. **Xinglin Wu:** Writing – review & editing, Writing – original draft, Methodology, Data curation. **Xiwen Luo:** Visualization, Validation. **Mingsheng Ma:** Methodology, Investigation. **Xi Yang:** Methodology, Investigation. **Zhiyong Zhang:** Methodology, Investigation. **Yunfei An:** Methodology, Investigation. **Xiaodong Zhao:** Methodology, Investigation. **Hongmei Song:** Methodology, Investigation. **Xuemei Tang:** Project administration, Methodology, Investigation, Funding acquisition.

## Ethics declaration

This study has been approved by the Ethics Committee of Children's Hospital of Chongqing Medical University (2021 Lun Shen (Lin Yan) No. 21). The participants provided informed consent prior to participating in the study.

## Availability of data and materials

Data are accessible upon reasonable request. All relevant data can be acquired by reaching out to the corresponding author.

## Funding

This research received funding from the 10.13039/501100012166National Key R&D Program of China (No. 2021YFC2702003).

## Conflict of interests

Xiaodong Zhao is the member of Genes & Diseases Editorial Board. To minimize bias, he/she was excluded from all editorial decision-making related to the acceptance of this article for publication. The remaining authors declare no conflict of interests.
