# Supplementary material for: CD14+CXCL10+ monocytes are associated with peripheral immune network alterations in systemic juvenile idiopathic arthritis: From multiple centers
Source: Genes Dis. 2025 Nov 19;13(4):101942. doi: 10.1016/j.gendis.2025.101942 (PMC13091345; doi:10.1016/j.gendis.2025.101942)
Supplement: Multimedia component 1 [file mmc1.docx]

**Supplementary Methods**

**Data processing and cell clustering of individual cases**

Preprocessed gene expression matrices from each sample were independently analyzed using RStudio (v4.0.2) and the Seurat package (v4.1.0) (16). Initial quality control excluded ribosomal genes, genes expressed in fewer than three cells, and cells expressing fewer than 200 genes. And cells with >16% mitochondrial gene content, <3% ribosomal gene content, or >0% hemoglobin gene content were considered low quality and excluded from further analysis (17).

Monocytes were identified based on high expression of LYZ, FCN1, AIF1, and S100A12; NK cells by GNLY, KLRD1, NKG7, KLRB1, and KLRK1; B cells by CD79A, MS4A1, MZB1, and JCHAIN; megakaryocytes by ITGA2B and GP9; dendritic cells by TMPO and GIMAP4; and T cells by CD3E, TCF7, RACK1, IL7R, and IFITM1. Cluster identities were inferred based on the expression of characteristic markers.

**Data integration with batch effect collection**

To normalize and integrate multi-sample scRNA-seq datasets, we applied SCTransform-based normalization followed by Harmony batch correction. Specifically, each dataset was normalized using SCTransform with regularized negative binomial regression, and the top variable features were used for PCA reduction (18). Batch effects across samples (denoted by orig.ident) were corrected using the Harmony algorithm, yielding a shared low-dimensional representation. Following Harmony integration, we performed UMAP (RunUMAP(reduction = "harmony")) (19). Graph-based clustering was conducted using the FindNeighbors and FindClusters functions based on the first 20 Harmony-corrected principal components. To determine the optimal clustering resolution, we tested a range of resolutions (0.1–1.0) and evaluated the clustering structure using Clustree. Based on visual inspection of UMAP plots and cluster tree topology, we selected resolution = 0.9 for downstream cell population definition. Clusters with low unique feature counts or high mitochondrial content were excluded as low-quality or apoptotic cells. All visualizations were generated using DimPlot.

**Single cell tissue preference assessment**

The Ro/e were defined as the ratio of the observed frequency of a meta-cluster in a given tissue to its expected frequency under a uniform distribution. A Ro/e > 1 indicates over-representation of the meta-cluster in that tissue, whereas Ro/e < 1 indicates under-representation (20).

**DEG analysis**

DEG analysis was performed using FindMarkers or FindAllMarkers, applying a log fold change cutoff of 0.25 and requiring gene expression in at least 20% of cells. KEGG pathway enrichment analysis was performed on DEGs identified in specific clusters to explore their associated biological functions. Enrichment analysis was conducted using the KEGG database (21).

**Clustering and annotation of cell sub-populations**

To further dissect cellular heterogeneity within major immune populations, we performed subclustering for each major cell type. Extracted cells were independently processed through SCTransform normalization, variable gene selection, scaling, and dimensionality reduction using PCA and UMAP. Multiple clustering resolutions (0-1) were tested, and the optimal resolution was selected based on cluster number, UMAP distribution, and DEG profiles, ensuring biological interpretability without over-clustering. To characterize transcriptional programs, we performed gene set activity analysis using the AUCell algorithm. Gene sets were obtained from MSigDB, including HALLMARK, KEGG, and REACTOME. AUCell computed AUC scores per gene set per cell, indicating gene set activity. Differential enrichment between subclusters was assessed using the Wilcoxon rank-sum test, followed by Bonferroni correction. Significantly enriched gene sets (adjusted p < 0.05) were ranked by log₂ AUC fold change. To interpret results, we calculated Pearson correlations between the top 10 highly expressed genes and enriched gene sets in each subcluster. The genes most strongly correlated were used to functionally annotate or name subclusters, reflecting dominant regulatory or inflammatory programs (22).

**Trajectory analysis**

We performed cellular trajectory inference on the integrated scRNA-seq dataset using multiple computational frameworks, including CytoTRACE (23), Monocle2 (16), Monocle3 (24), Slingshot (16), and VECTOR (25). For Monocle2, we first constructed a CellDataSet (CDS) object using the newCellDataSet() function, followed by size factor estimation, dispersion calculation, and variance-stabilizing transformation. Highly variable genes were selected for dimensionality reduction using the reduceDimension() function with method = "DDRTree", and trajectory inference was performed using orderCells(). Cells were ordered along the pseudotime axis, and trajectory branches were visualized using the plot_cell_trajectory() function. For Monocle3, we applied the preprocess_cds() function with num_dim = 100 to normalize the dataset and perform dimensionality reduction. Cells were clustered using cluster_cells(), and UMAP-based embeddings were generated via reduce_dimension(). The learn_graph() function was used to construct principal graphs within each cluster, and pseudotime trajectories were visualized with plot_cells() colored by pseudotime values. For Slingshot, UMAP embeddings of monocyte populations were used as input to infer lineage relationships and developmental trajectories across transcriptionally distinct clusters. To complement these approaches, we also applied VECTOR, a novel R package recently published in Cell Reports, to infer developmental directionality. VECTOR constructs vector fields in UMAP space based on network distances between cells, thereby predicting cell-state transitions without relying on spliced/unspliced transcript quantification or BAM-to-loom format conversion. This significantly streamlines the analysis while preserving high concordance with RNA velocity models and enhancing computational efficiency. Lastly, we applied CytoTRACE, a computational framework that predicts cell developmental potential based on transcriptional diversity, without requiring prior lineage annotation or pseudotime ordering. Gene expression matrices were input into the CytoTRACE pipeline using default parameters, and the inferred CytoTRACE scores were used to rank cells from highest to lowest developmental potential, providing an orthogonal measure of cellular plasticity complementary to trajectory-based pseudotime inference.

**Cell-cell communication and ligand–target regulatory analysis**

Intercellular communication networks were inferred using the CellChat R package (v1.6.1) (26). Normalized gene expression data and cell type annotations from the Seurat object were imported into CellChat using the createCellChat() function. Significant ligand–receptor interactions were identified using identifyOverExpressedGenes(), identifyOverExpressedInteractions(), and computeCommunProb(). Interaction strengths and counts were compared across groups using the compareInteractions() function. Visualization was performed using netVisual_circle() to display global communication networks, netVisual_bubble() for specific ligand–receptor pairs, and pheatmap to show the distribution of interaction frequencies among cell types.

To further dissect ligand-driven regulation of proinflammatory programs in CD14+CXCL10+ monocyte, we employed NicheNet to predict active ligands from potential sender cell types and their effects on target gene expression in monocytes (27). Differentially expressed genes were identified by comparing CD14+CXCL10+ monocyte in disease versus control samples, and the top 100 upregulated genes (excluding mitochondrial and sex chromosome-associated genes) were selected as the target gene set. Ligands with predicted receptor expression in monocytes and a gene regulatory potential were screened using NicheNet’s built-in ligand–target network. Sender and receiver cell identities were assigned based on annotated Seurat clusters, and ligand activity scores were computed using the nichenet_seuratobj_aggregate() workflow. Predicted ligand–target links were visualized using pheatmap, ranking ligands by their regulatory intensity on the selected inflammatory gene program.

**Immune-related Gene Set Enrichment Analysis (irGSEA)**

To characterize immune pathway activity at single-cell resolution, we performed immune-related gene set enrichment analysis (irGSEA) using the irGSEA R package (v1.0.1) (28). Single-cell transcriptomic data from sJIA patients were processed using the Seurat pipeline, and major immune cell types were annotated. Gene set collections were retrieved from the MSigDB (v2023.2.Hs), including KEGG, Reactome, and Hallmark pathways. Enrichment scores were computed for each cell using multiple single-cell GSEA methods, including AUCell, UCell, and singscore, with additional methods (ssGSEA, viper, JASMINE) applied for cross-validation. The irGSEA.score() function was used with custom = TRUE and geneset = msigdb.h, utilizing 30 computational threads. Cells were grouped based on UBE2D1 expression levels (high vs. low) or by main immune cell type annotation. To identify robust gene sets consistently differentially enriched across scoring methods, robust rank aggregation (RRA) was performed using the irGSEA.integrate() function.

**RNA-Seq data for Chongqing cohort**

Total RNA was extracted from JIA and control PBMCs using TRIzol Reagent (Invitrogen, Cat. No. 15596026) according to the protocol by Chomczynski et al. Following extraction, DNase I treatment was applied to eliminate residual genomic DNA. RNA purity was assessed by measuring the A260/A280 ratio using a NanoDrop OneC spectrophotometer (Thermo Fisher Scientific), and RNA integrity was verified by 1.5% agarose gel electrophoresis. Final quantification was performed with the Qubit 3.0 Fluorometer using the Qubit RNA Broad Range Assay Kit (Life Technologies, Q10210). For stranded mRNA library construction, 2 μg of total RNA was used with the KCTM Stranded mRNA Library Prep Kit for Illumina® (Cat. No. DR08402, Wuhan Seqhealth Co., Ltd., China), following the manufacturer’s instructions. PCR-amplified fragments (200–500 bp) were enriched and quantified prior to high-throughput sequencing on the DNBSEQ-T7 platform (MGI Tech Co., Ltd., China) using paired-end 150 bp (PE150) mode. Raw sequencing reads were pre-processed using Trimmomatic (v0.36) to remove low-quality bases and adaptor sequences. Clean reads were aligned to the human reference genome (Ensembl GRCh38) using the STAR aligner (v2.5.3a) with default parameters. Gene-level counts were obtained using featureCounts (Subread v1.5.1, Bioconductor) by quantifying reads mapped to exon regions of annotated genes. The resulting raw count matrix was used for subsequent downstream analyses.

**Analysis of signatures of monocyte subsets in the Chongqing cohort**

Bayesian deconvolution was conducted using the BayesPrism R package (v1.1.0) to estimate immune cell type proportions from bulk RNA-seq data of PBMCs, including samples from patients with sJIA, non-sJIA and controls (29). Raw gene expression count matrices were used as input. The reference matrix was constructed from a previously annotated single-cell RNA-seq dataset of PBMCs. Top marker genes for each immune cell type, including CD14+CXCL10+ monocyte, were selected to define cell-type-specific expression profiles. Cell type annotations were provided via the cell_meta metadata file, and the key parameter was set to NULL to allow all cell types to contribute equally to the model. An automated wrapper function (afBayesPrism()) was used to batch-process all bulk RNA-seq samples. To enhance accuracy and computational efficiency, only protein-coding genes were retained using the select.gene.type() function. The BayesPrism workflow was run using new.prism() and run.prism() with 15 CPU cores (n.cores = 15). The posterior mean estimates of cell type fractions (theta matrix) were extracted using get.fraction() with which.theta = "final", and stored for downstream analysis. After deconvolution, posterior mean estimates of CD14+CXCL10+ monocyte fractions were extracted and subjected to downstream analyses. To investigate their clinical relevance, Spearman rank correlation was performed between the deconvolution scores and inflammatory and disease activity marker. Correlation results were visualized using the corrplot R package, with dot size and color representing the magnitude and direction of the correlation coefficient. Moreover, patient responses to Canakinumab in the Chongqing cohort were documented, and signature scores were compared between responders and non-responders using the Wilcoxon test.

**Flow cytometry and cell sorting**

PBMCs from patients with sJIA were isolated by density gradient centrifugation and cryopreserved. Upon use, cells were thawed, washed with cold PBS, and treated with DNase I (1 mg/mL, Roche) to prevent aggregation. Red blood cells were lysed using RBC lysis buffer (Absin), and cells were filtered through a 70 μm strainer. Fc receptors were blocked using an FcR blocking reagent (Absin), followed by viability staining with Fixable Viability Stain 780 (BD, Cat# 565388) at 4 °C for 30 minutes in the dark. Surface markers were stained using CD14-PE-Cy7 (BD, 557742), CD11b-FITC (Absin, Abs180027), CD16-BV510 (BD, 563830), and CD66b-V450 (BD, 561649). After surface staining, cells were fixed and permeabilized using the Fixation/Permeabilization Kit (BD, 554714) and stained intracellularly with anti-CXCL10-PE (BioLegend, 519504) following a 4-hour Brefeldin A (Absin) incubation. Samples were acquired on a BD LSRFortessa X-20 and analyzed using FlowJo v10.8.1. Gating excluded debris, dead cells, and CD66b+ neutrophils; CXCL10 expression was quantified within the CD14+ monocyte population gated from live CD66b⁻CD45+ cells.

**Western blotting**

PBMCs were isolated from human peripheral blood using a commercial PBMC isolation solution and cultured in RPMI 1640 medium supplemented with 10% fetal bovine serum (FBS) and 1% penicillin-streptomycin. Where indicated, cells were transfected using HighGene transfection reagent following the manufacturer's protocol. After treatment, total protein was extracted from PBMCs using RIPA lysis buffer containing protease inhibitors, and concentrations were determined using a bicinchoninic acid (BCA) assay. Equal amounts of protein were denatured at 100 °C for 10 minutes, separated by 10% SDS-PAGE, and transferred to PVDF membranes. Membranes were blocked with 5% bovine serum albumin (BSA) for 1 hour at room temperature and incubated overnight at 4 °C with primary antibodies against UBE2D1 (1:1000) and β-actin (1:5000, used as a loading control). After washing, membranes were incubated with HRP-conjugated goat anti-rabbit or goat anti-mouse IgG secondary antibodies at room temperature for 1 hour.

**Cytokine and LPS stimulation followed by qPCR and ELISA**

THP-1 cells were cultured in RPMI 1640 medium supplemented with 10% fetal bovine serum and 1% penicillin-streptomycin under standard conditions. Cells were seeded in 6-well plates and stimulated with lipopolysaccharide (LPS, 100 ng/mL), IL-6 (20 ng/mL), IL-1β (10 ng/mL), or TNF-α (10 ng/mL) for 0, 12, 24, or 48 hours. Untreated cells served as controls. At each time point, cell pellets were collected for RNA extraction, and supernatants were harvested for protein detection. Total RNA was extracted using TRIzol reagent, and cDNA was synthesized using a reverse transcription kit. Quantitative PCR was performed using a SYBR Green master mix on a CFX96 real-time PCR system, with β-actin as the reference gene. The 2⁻ΔΔCt method was used to calculate relative CXCL10 and UBE2D1 mRNA expression, normalized to the 0 h control. CXCL10 protein levels in culture supernatants were quantified by ELISA using commercial kits according to the manufacturers’ instructions. Absorbance was measured at 450 nm, and concentrations were calculated based on standard curves. All conditions were tested in triplicate.

UBE2D1:

Primer F: 5'AGCGCATATCAAGGTGGAG3'

Primer R: 5'AGAGCTGGTGACCATTGTG3'

CXCL10:

Primer F: 5'TGGCATTCAAGGAGTACCTC3'

Primer R: 5'TTGTAGCAATGATCTCAACACG3'

**CCL4 stimulation and gene expression analysis in THP-1 cells**

THP-1 cells were cultured in RPMI 1640 medium supplemented with 10% fetal bovine serum and 1% penicillin-streptomycin under standard conditions (37 °C, 5% CO₂). Cells were seeded in 6-well plates and stimulated with CCL4 (20 ng/mL) and LPS for 24 hours, while LPS cells served as controls. After stimulation, total RNA was extracted using TRIzol reagent (Invitrogen, USA), and cDNA was synthesized using a cDNA Reverse Transcription Kit (Roche, Switzerland). IL-1β and UBE2D1 mRNA expression levels were quantified by real-time PCR using a SYBR Green master mix on a CFX96 Real-Time PCR System (Bio-Rad). β-actin was used as an internal reference gene. Relative gene expression was calculated using the 2⁻ΔΔCt method, and values were normalized to the control group. Each condition was analyzed in triplicate, and statistical significance was evaluated using an unpaired two-tailed Student’s t-test.

**UBE2D1 siRNA and plasmid transfection in THP-1 cells**

THP-1 cells were transfected with UBE2D1-specific siRNA or UBE2D1 overexpression plasmid using HighGene transfection reagent (commercial supplier) according to the manufacturer’s instructions. Briefly, 4 μg of plasmid DNA or 100 pmol of UBE2D1 siRNA was diluted in 200 μL of serum-free DMEM and mixed thoroughly by pipetting. Subsequently, 6 μL of HighGene reagent was added to the mixture, followed by gentle pipetting to ensure complete complex formation. The transfection mixture was added dropwise to cells seeded in 6-well plates, and the plate was gently rocked to distribute the complex evenly. After 4–6 hours of incubation at 37 °C, half of the medium was replaced with fresh complete RPMI-1640 medium supplemented with 10% fetal bovine serum and 1% penicillin-streptomycin. After 24 hours of transfection, cells were harvested for transcriptomic profiling. RNA extraction, library preparation, and RNA sequencing were conducted by Biaoguan shengwu (China) using standard stranded mRNA-seq protocols. Raw sequencing data were used for downstream differential expression and enrichment analyses.

**Gene set enrichment analysis (GSEA)**

To investigate the biological pathways associated with different levels of UBE2D1 expression following transfection, samples were divided into three groups based on transfection conditions: low expression. Gene expression data were extracted from the transfection-associated expression matrix. For GSEA, the low and high expression groups were compared by calculating the average expression of each gene, and the logFC between the two groups was used as the ranking metric. Genes were ordered by logFC to construct a preranked gene list. Gene set annotations were derived from the MSigDB C2 canonical pathway collection. Enrichment analysis was performed using the GSEA function from the clusterProfiler R package. Significantly enriched pathways were defined as those with p-value < 0.05, and the normalized enrichment score (NES) was used to determine directionality. The top five pathways enriched in both the high and low expression groups were visualized using the gseaplot2() function from the enrichplot package.

**Ube2d1 knockout mice and genotyping**

Ube2d1 conventional knockout mice (C57BL/6JCya-Ube2d1<sup>em1/Cya</sup>) were generated using CRISPR/Cas9 technology and obtained from GemPharmatech Co., Ltd. (China). The gene targeting strategy involved deletion of exons 2 to 8, encompassing the full coding sequence of the Ube2d1 gene. Founder mice were confirmed by PCR and Sanger sequencing, revealing a 6863 bp deletion at the target locus. Heterozygous (Ube2d1<sup>+/−</sup>) F1 mice were intercrossed to generate wild-type (WT), heterozygous (Het), and homozygous knockout (KO) offspring. Genotyping was performed using the following primer sets:

F1 primer: 5′-TAAGAGCAGTTCCCCTAAGCCT-3′.

R1 primer: 5′-ACACAGACATTTAACCTGCAGAGA-3′.

R2 primer: 5′-CCAAGGATCTGTGCAAAGAGAAG-3′.

PCR amplification produced: 570 bp band indicating knockout allele. 638 bp band indicating wild-type allele. Mice were housed in specific-pathogen-free (SPF) conditions on a 12-hour light/dark cycle with free access to food and water. Ambient temperature was maintained at 22–24 °C and humidity at 45–55%.

**Ube2d1 knockout mice and collagen antibody-induced arthritis (CAIA) model**

To induce arthritis, 8-week-old male mice were subjected to the collagen antibody-induced arthritis (CAIA) model. Mice were injected intravenously with 5 mg of ArthritoMab™ anti-collagen type II monoclonal antibody cocktail on day 0, followed by an intraperitoneal injection of 50 μg lipopolysaccharide (LPS) on day 3 to enhance disease induction. Clinical signs of arthritis, including paw swelling and joint rigidity, were monitored daily using a standardized scoring system. Mice were sacrificed on day 14 for downstream analyses including histopathology, RNA extraction, and cytokine quantification.

Arthritis severity was scored every two days by two independent observers using a 0–4 scale: 0, no redness or swelling; 1, knuckle swelling; 2, mild swelling of the ankle or wrist; 3, pronounced swelling of the entire paw; 4, joint stiffness or deformity.

**RNA sequencing of mouse spleen samples**

Spleen tissues were collected from mice on day 14 following CAIA induction. Total RNA was extracted from fresh spleen samples and subjected to quality control and library preparation by Meister Biotechnology Co., Ltd. (China). RNA integrity was assessed using an Agilent Bioanalyzer 2100 system, and sequencing libraries were constructed using a stranded mRNA-seq protocol. Libraries were sequenced on the Illumina NovaSeq platform with paired-end 150 bp (PE150) reads. Raw sequencing data were subjected to quality control, and clean reads were aligned to the mouse reference genome (GRCm39) using STAR (v2.5.3a). Gene-level read counts were quantified using featureCounts (Subread v1.5.1), and the resulting raw count matrix was used for downstream differential expression and enrichment analyses.

**Cytokine profiling in peripheral blood of CAIA mice**

Peripheral blood samples were collected postmortem from CAIA mice on day 14 after arthritis induction. Mice were euthanized, and blood was obtained via cardiac puncture using sterile syringes. After clotting at room temperature for 30 minutes, samples were centrifuged at 3000 rpm for 10 minutes at 4 °C to isolate serum. Cytokine levels in the serum were measured using the RayBiotech® Mouse Cytokine Antibody Array platform, and all experimental procedures, including sample incubation, detection, and data analysis, were performed by Zeheng Ruiboa Biotechnology Co., Ltd. (Chengdu, China). The array enabled simultaneous detection of multiple cytokines, including IL-1β, IL-6, TNF-α, IFN-γ, IL-10, and CXCL10. Signal intensities were acquired via chemiluminescence and analyzed using the RayBio® Q Analyzer software. Data were normalized to internal positive controls, and all samples were measured in technical duplicates.

**Enzyme-linked immunosorbent assay in mouse joints**

The protein levels of key inflammasome and innate immune signaling components in mouse joint tissues were quantified using ELISA kits provided by Sanying Biotechnology Co., Ltd. (Shanghai, China). The following mouse-specific ELISA kits were used: NLRP1, NLRP3, NLRC4, NOD1, NOD2, and IκB-α, according to the manufacturer’s protocols. Joint tissues were harvested from euthanized CAIA mice on day 14 post-induction and homogenized in cold PBS containing protease inhibitors. After centrifugation at 12,000 × g for 10 minutes at 4 °C, supernatants were collected for ELISA analysis. All reagents and samples were brought to room temperature before use. 100 μL of sample or standard was added to each well of the pre-coated ELISA plates and incubated at 37 °C for 90 minutes. After incubation with biotinylated detection antibodies and HRP-conjugated reagents, substrate solution was added, and the reaction was stopped using stop buffer. Absorbance was measured at 450 nm using a microplate reader, and cytokine concentrations were calculated from standard curves.

**Micro-CT Analysis**

Following euthanasia by cervical dislocation, the left hind paw of each mouse was immediately harvested and fixed in 4% paraformaldehyde at room temperature for 24–48 hours. Fixed specimens were then transferred to the Oral Hospital of Chongqing Medical University for micro-computed tomography (Micro-CT) imaging. If immediate scanning was not feasible, samples were temporarily stored in 70% ethanol after fixation. Scanning was performed using a high-resolution Micro-CT system, and three-dimensional reconstruction as well as structural analysis of the hind paw joints were carried out to evaluate bone erosion and joint damage. Quantitative assessment of bone morphology, including measurements of bone mineral density (BMD) and bone volume (BV), was performed using the system’s built-in analysis software.

**Multiplex Immunofluorescence Staining**

Multiplex immunofluorescence was performed to evaluate immune cell subtypes and Cxcl10 expression in murine tissue sections using the TSA Plus Fluorescence Double Staining Kit (Product No. G1226-50T/100T, Servicebio). Formalin-fixed, paraffin-embedded (FFPE) sections were deparaffinized and rehydrated through graded ethanol series, followed by antigen retrieval in EDTA buffer via microwave treatment. Endogenous peroxidase activity was quenched with 3% hydrogen peroxide, and non-specific binding was blocked using 3% bovine serum albumin (BSA). Sections were incubated with primary antibodies overnight at 4 °C, followed by HRP-conjugated secondary antibodies and tyramide signal amplification (TSA) using iF488 and iF555 fluorophores. Between cycles, microwave-based antibody stripping was applied to remove previously bound antibodies. Nuclei were counterstained with DAPI. Prior to mounting with antifade medium, autofluorescence was quenched. Fluorescence images were acquired using the PanoBrain multiplex imaging system (Servicebio), and signal distribution was quantified across target regions.

**Figure S1** Composition of immune cell populations in the sJIA and control groups. (Left) Bar plot showing the relative proportions of immune cell types in the sJIA group and control groups. (Right) Comparison of immune cell composition across the sJIA-internal, sJIA-external, sJIA-after-treatment, and control groups.

**Figure S2** Differential proportions of monocytes across the Chongqing Clinical Dataset (**P* < 0.05, ***P* < 0.01, ****P* < 0.001, and *****P* < 0.0001). Abbreviations: oJIA, oligoarticular juvenile idiopathic arthritis; pJIA, polyarticular juvenile idiopathic arthritis; ERA, enthesitis-related arthritis; sJIA, systemic juvenile idiopathic arthritis; Unnamed JIA, undifferentiated juvenile idiopathic arthritis.

**Figure S3** Single-cell atlas of PBMCs from patients with sJIA and controls. **(A)** Correlation between monocyte signature scores and IL-1β secretion in sJIA and non-sJIA. **(B)** KEGG enrichment analysis of monocytes. **(C)** An illustration highlighting the enrichment *P* values for selected GO biological pathways of differentially expressed genes (DEGs) in each cell type, color-coded for each cell.

**Figure S4** KEGG enrichment analysis in different cells.

**Figure S5** Correlation between the top 10 genes of monocyte subpopulation expression and biological functional scores.

**Figure S6** Stacked bar plots showing the relative abundance of monocyte subtypes in each group.

**Figure S7** Correlation between the top 10 genes of CD14^+^CXCL10^+^ monocyte expression and biological functional scores (functional enrichment activity scores were derived from AUCell analysis with MSigDB collections (HALLMARK, KEGG, REACTOME)). Correlations with the top-expressed genes were assessed via Spearman’s rank test).

**Figure S8** ROC curve illustrating the diagnostic performance of CD14^+^CXCL10^+^ monocytes for distinguishing sJIA.

**Figure S9** Subgroup annotation in T, B, and NK cells.

**Figure S10** Sankey plots depicting outgoing (left) and incoming (right) communication patterns. Cell groups are connected to their dominant signaling patterns (middle) and further to specific signaling pathways (right side).

**Figure S11** Differential ligand–receptor signaling patterns in CD14^+^CXCL10^+^ monocytes.

**Figure S12** Inference of ligand–target regulatory networks in CD14^+^CXCL10^+^ monocytes using NicheNet in treatment.

**Figure S13** Analysis of monocyte2 developmental trajectories in sJIA. **(A)** Dynamic gene expression modules along the pseudotime trajectory and associated functional enrichment. **(B)** Pseudotime trajectory analysis reveals the dynamic differentiation path of monocyte subsets. (Left) Trajectory inference of monocyte subpopulations was performed using monocle2. The cells are arranged along a pseudotime trajectory. The cells are color-coded according to pseudotime (top) and cell subtypes (bottom), revealing a branched differentiation path. (Right) Density plots display the distribution of each monocyte subtype along pseudotime.

**Figure S14** Overlapping DEG genes between the internal and external sJIA datasets.

**Figure S15** KEGG pathway alterations in CD14^+^CXCL10^+^ monocyte DEGs in the external sJIA set, the internal sJIA set and post-treatment.

**Figure S16** Functional enrichment analysis of the UBE2D1 expression phenotype. **(A)** Functional enrichment analysis of the UBE2D1 expression phenotype (UBE2D1-high group *vs.* control). **(B)** Functional enrichment analysis of the UBE2D1 expression phenotype (Control *vs.* UBE2D1-low group). **(C)** Functional enrichment analysis of the UBE2D1 expression phenotype (UBE2D1-high group *vs.* UBE2D1-low group).

**Figure S17** Proinflammatory stimuli induce UBE2D1 expression in monocytes.

**Figure 18** Correlation between Ube2d1 expression and inflammation-related markers in the CAIA model. **(A)** Scatter plot with marginal distributions between Ube2d1 and Cxcl10. **(B)** Box plot comparing NOD-like receptor signaling (NLR) pathway activity scores between Ube2d1 heterozygous knockout mice and wild-type controls.

**Figure S19** Comparative single-cell transcriptomic analysis in Kawasaki disease, BLAU, sepsis, and sJIA. **(A)** UMAP visualization of integrated scRNA-seq data in Kawasaki disease, BLAU, sepsis, and sJIA, with major immune cell types annotated. **(B)** Dot plot illustrating the expression of canonical marker genes used to annotate major immune cell populations. **(C)** Bar plot showing the relative proportions of immune cell types. **(D)** Annotations of monocyte subtypes in Kawasaki disease, BLAU, sepsis, and sJIA.

**Figure S20** CD14^+^CXCL10^+^ monocytes show disease-specific in Kawasaki disease, BLAU, sepsis, and sJIA. **(A)** CD14^+^CXCL10^+^ monocytes enriched in sepsis samples. **(B)** CD14^+^CXCL10^+^ monocytes enriched in Kawasaki disease (KD) samples. **(C)** CD14^+^CXCL10^+^ monocytes enriched in BLAU samples. **(D)** CD14^+^CXCL10^+^ monocytes enriched in BLAU samples. **(E)** Ratios of observed to expected proportions of CD14^+^CXCL10^+^ monocytes across the four diseases. **(F)** Violin plots displaying UBE2D1 expression levels in CD14^+^CXCL10^+^ monocytes from each disease cohort.
