# Supplementary figures and images for: CD14+CXCL10+ monocytes are associated with peripheral immune network alterations in systemic juvenile idiopathic arthritis: From multiple centers
Source: Genes Dis. 2025 Nov 19;13(4):101942. doi: 10.1016/j.gendis.2025.101942 (PMC13091345; doi:10.1016/j.gendis.2025.101942)

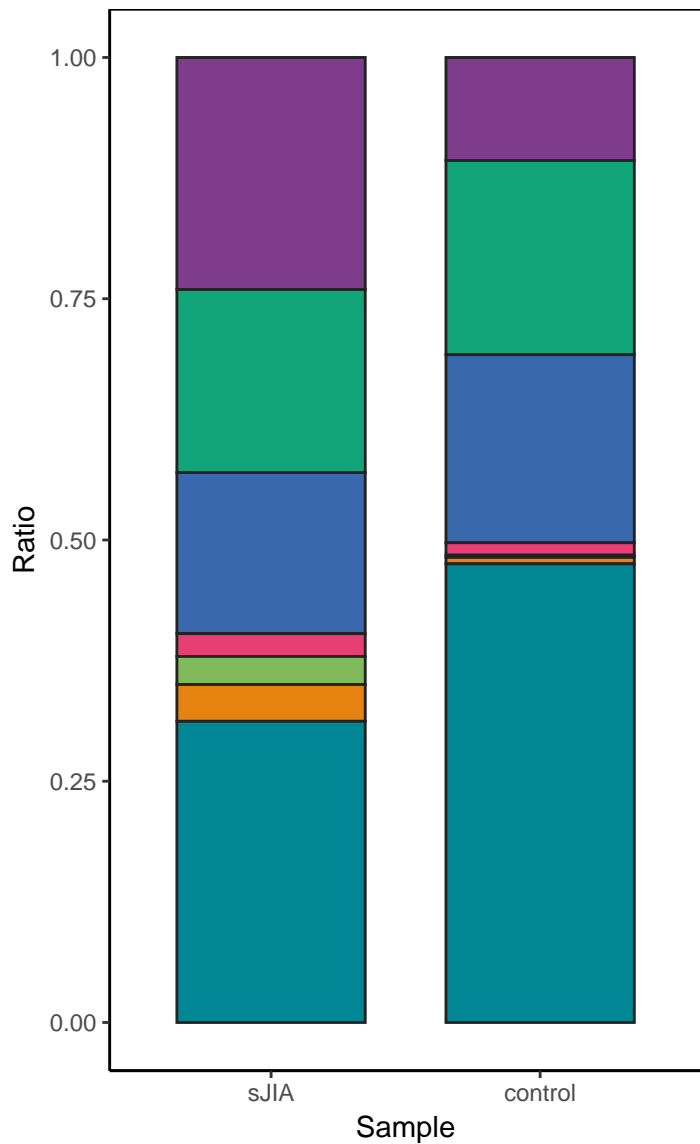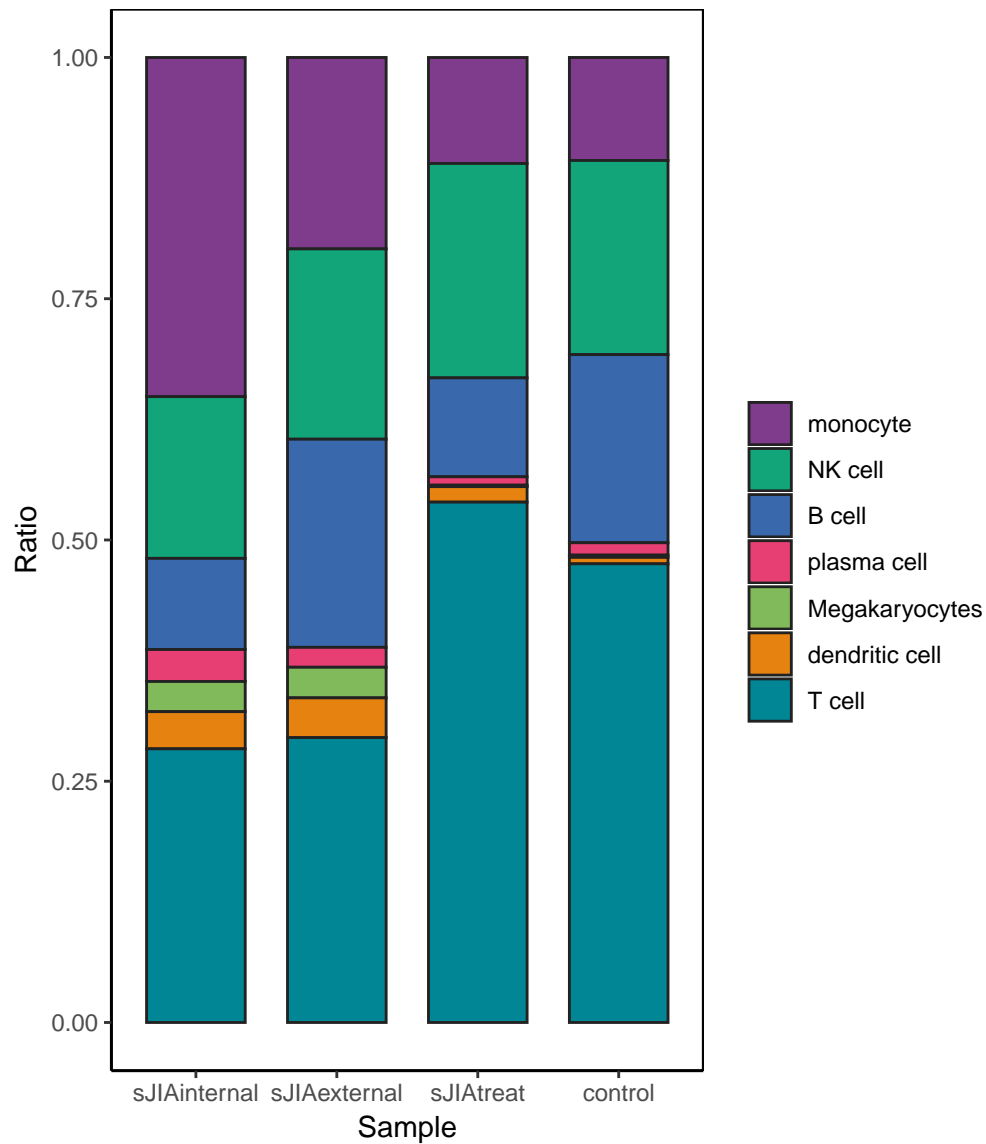

Supplement: Multimedia component 3 [file mmc3.pdf]

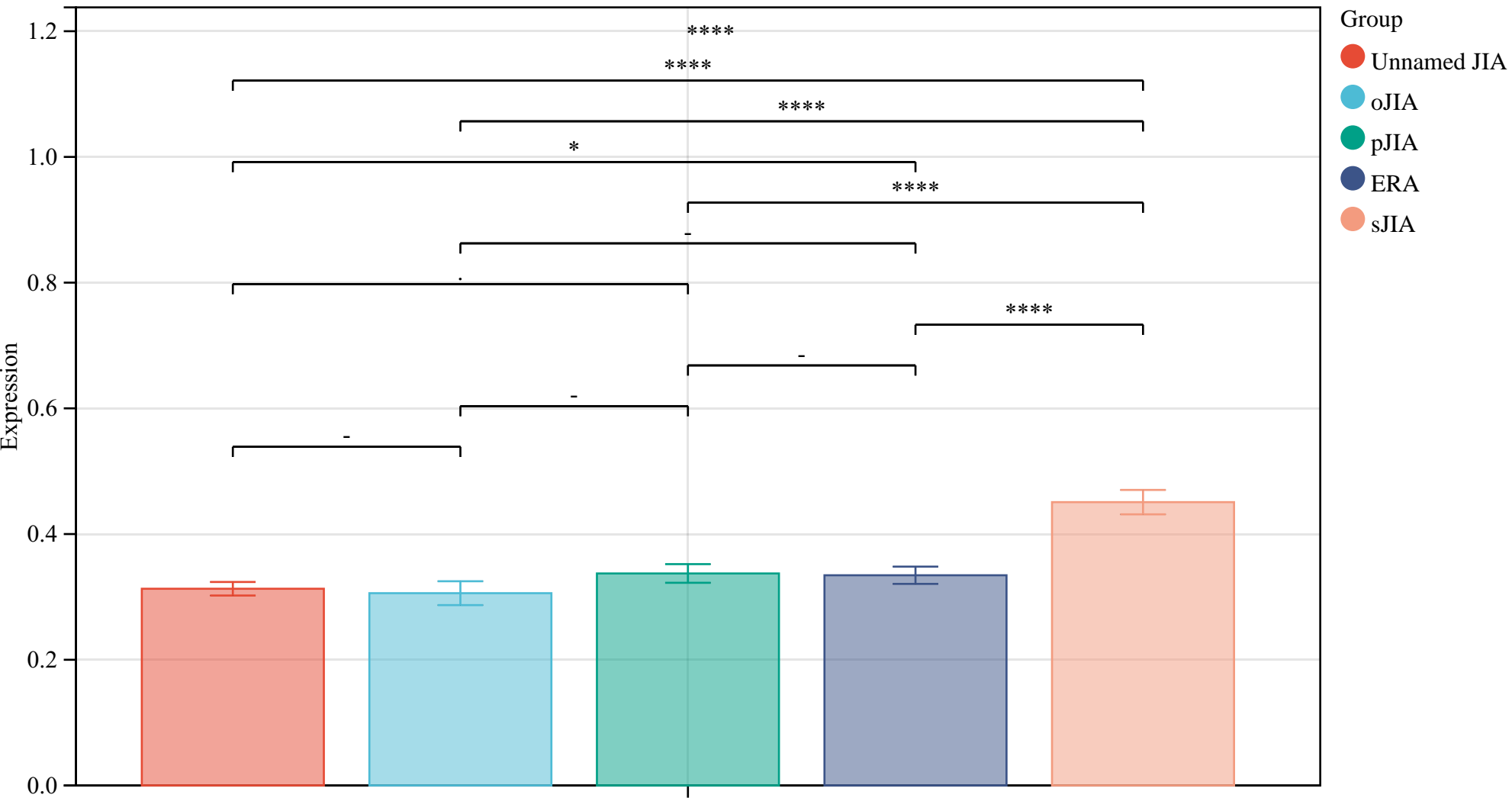

Supplement: Multimedia component 4 [file mmc4.pdf]

A

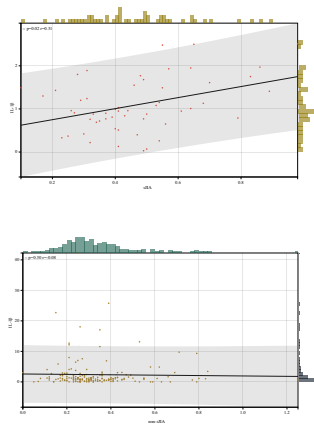

B

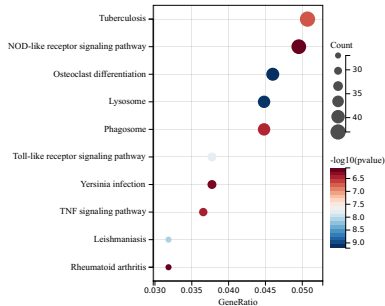

C

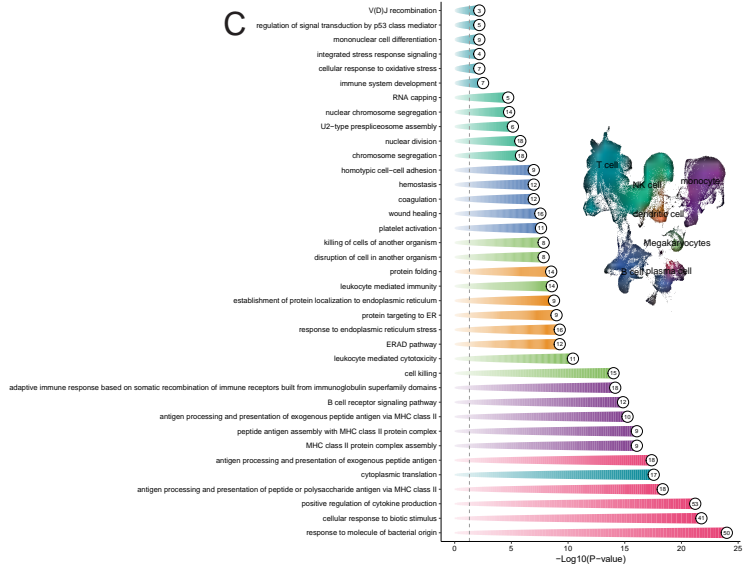

Supplement: Multimedia component 5 [file mmc5.pdf]

A

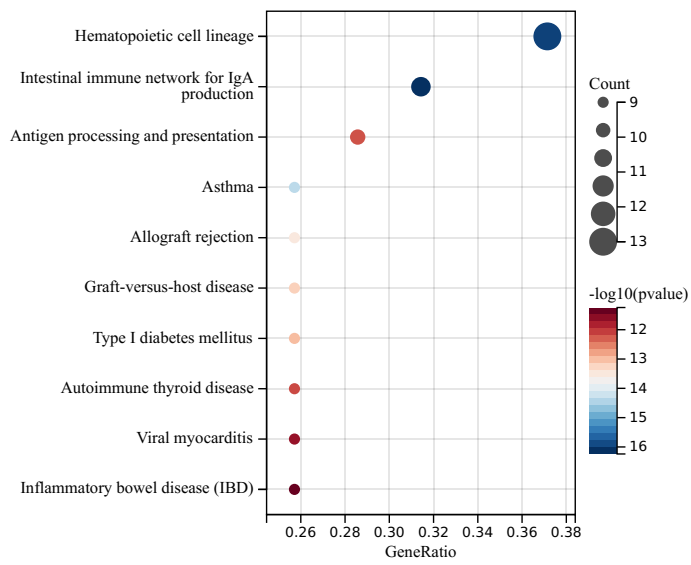

B

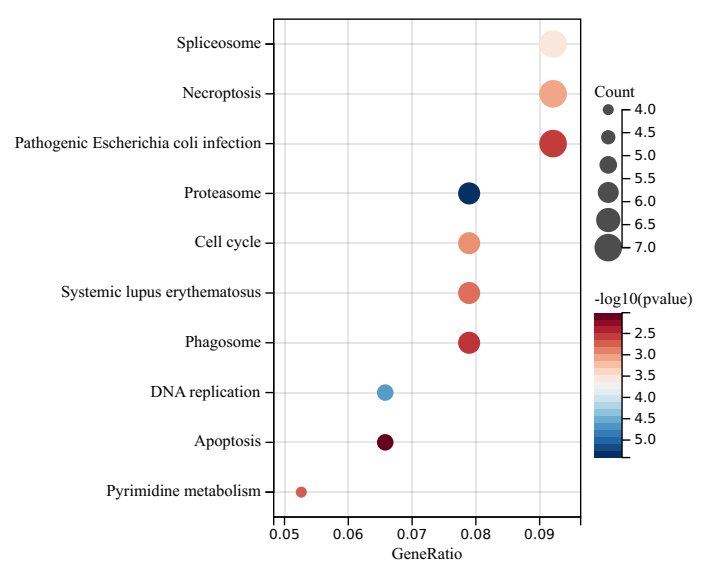

C

B cell

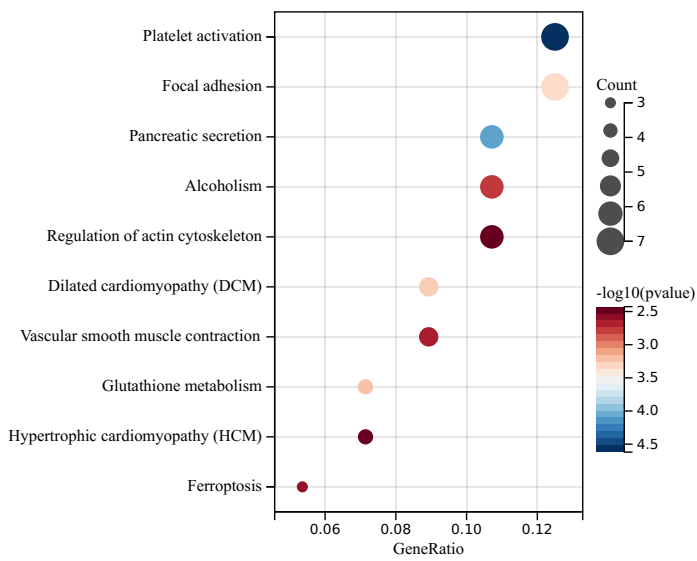

Megakaryocytes

D

DC cell

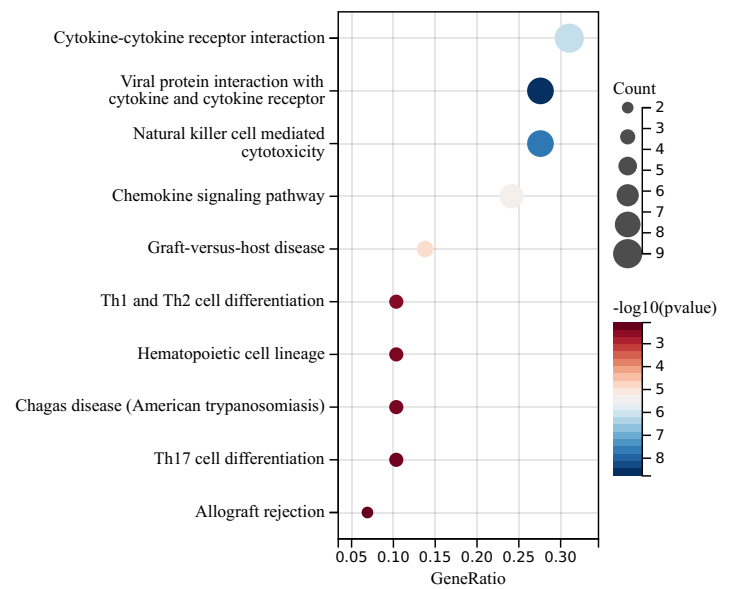

NK cell

E

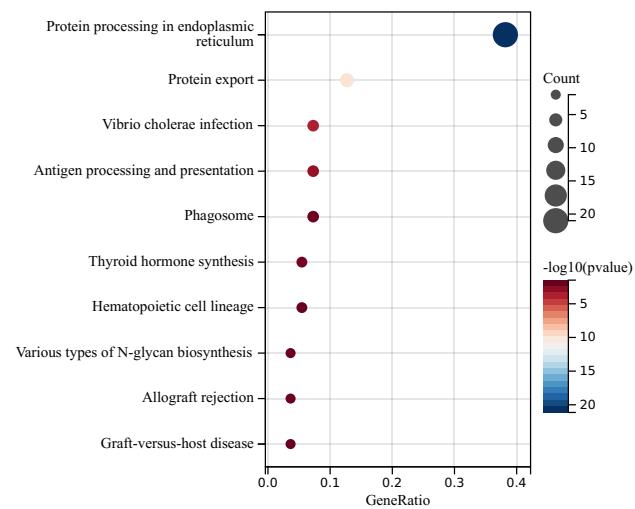

Plasma cell

F

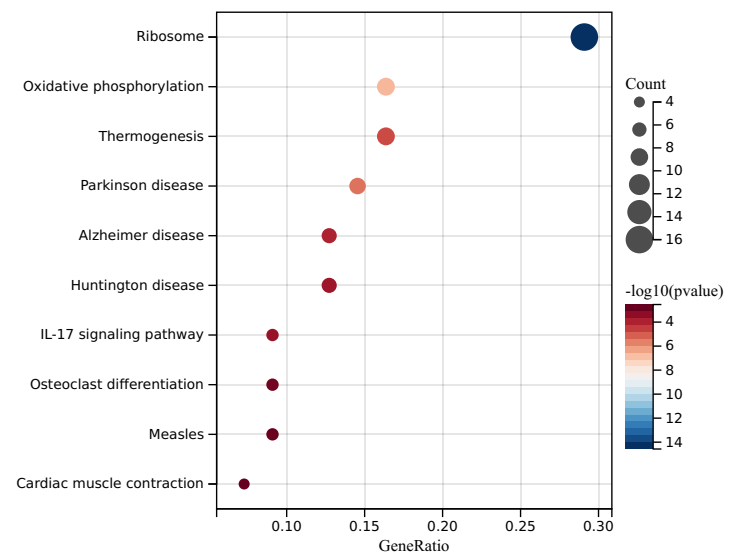

T cell

Supplement: Multimedia component 6 [file mmc6.pdf]

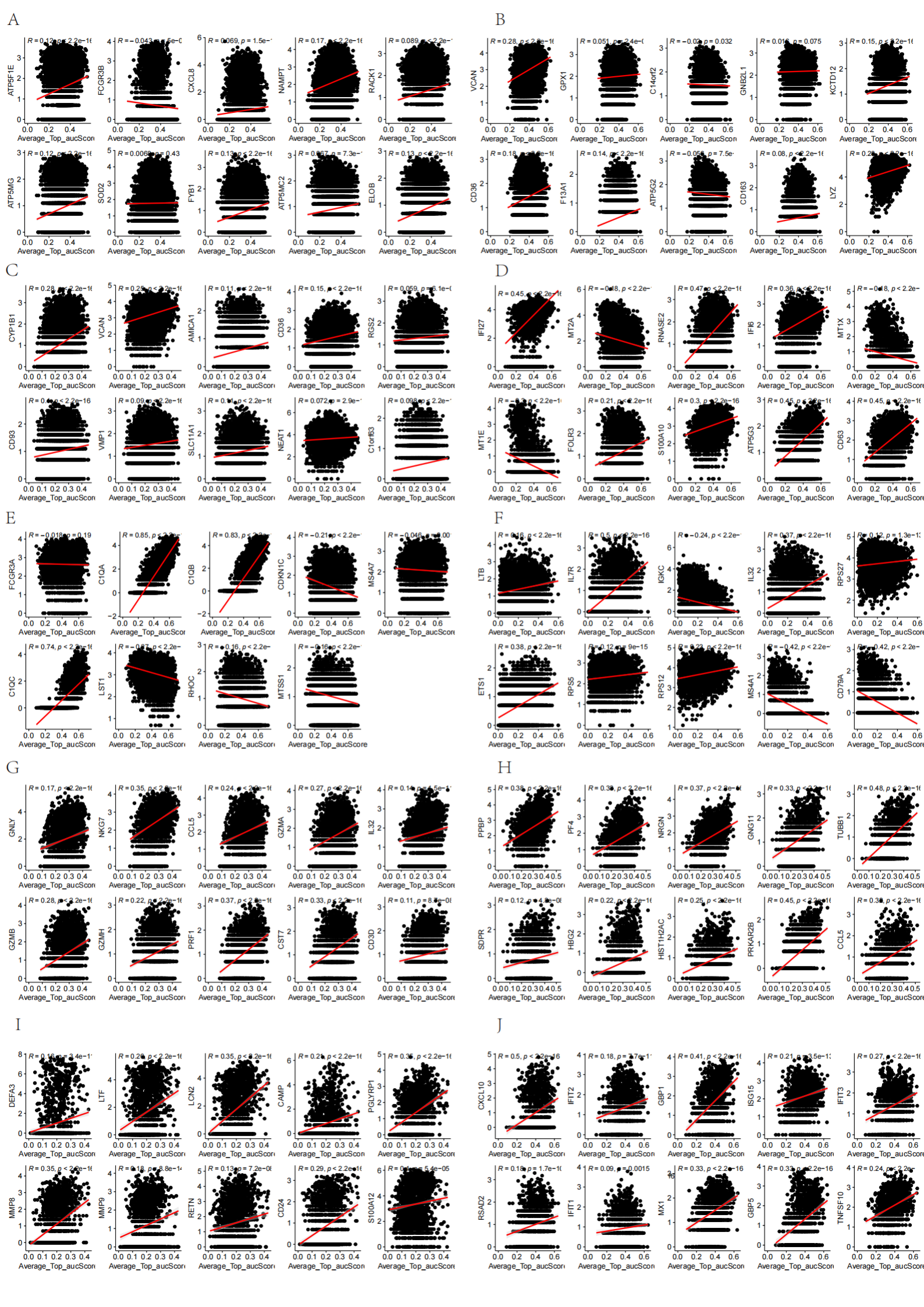

Supplement: Multimedia component 7 [file mmc7.pdf]

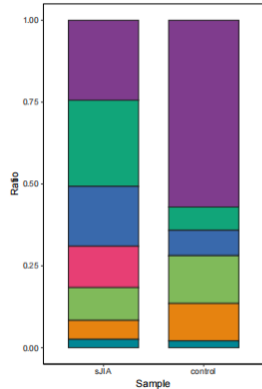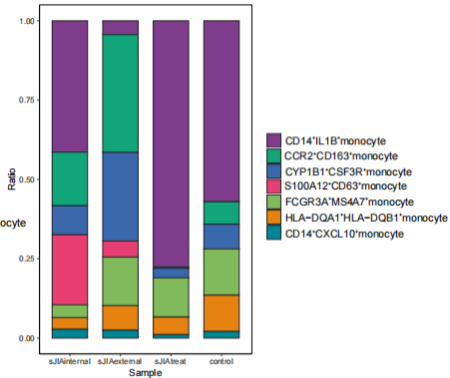

Supplement: Multimedia component 8 [file mmc8.pdf]

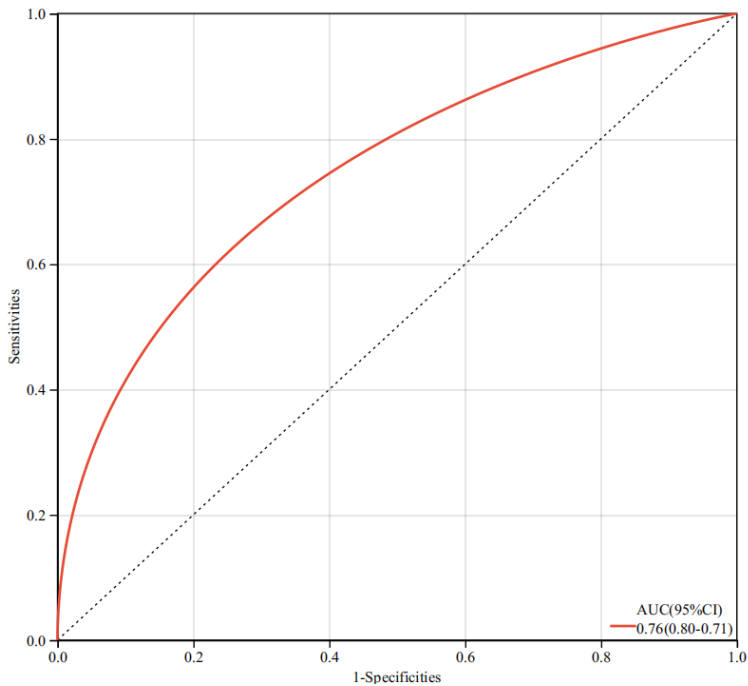

Supplement: Multimedia component 10 [file mmc10.pdf]

A

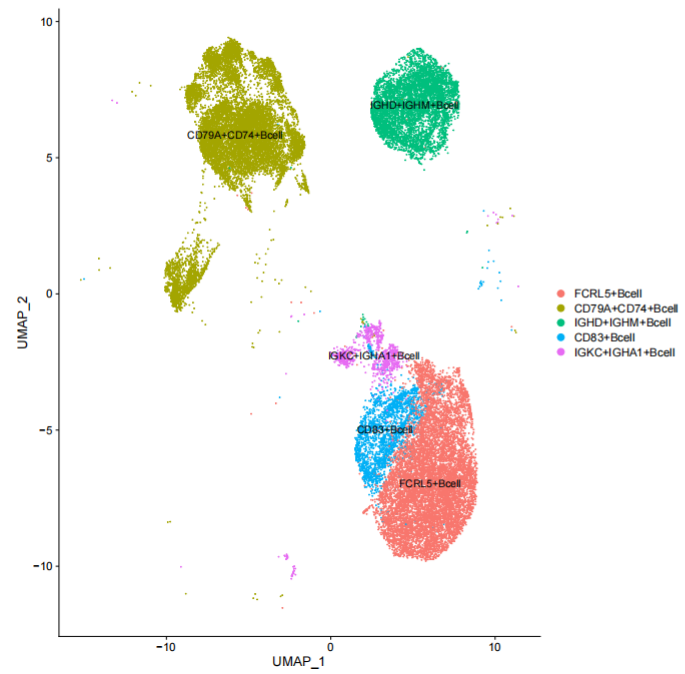

B

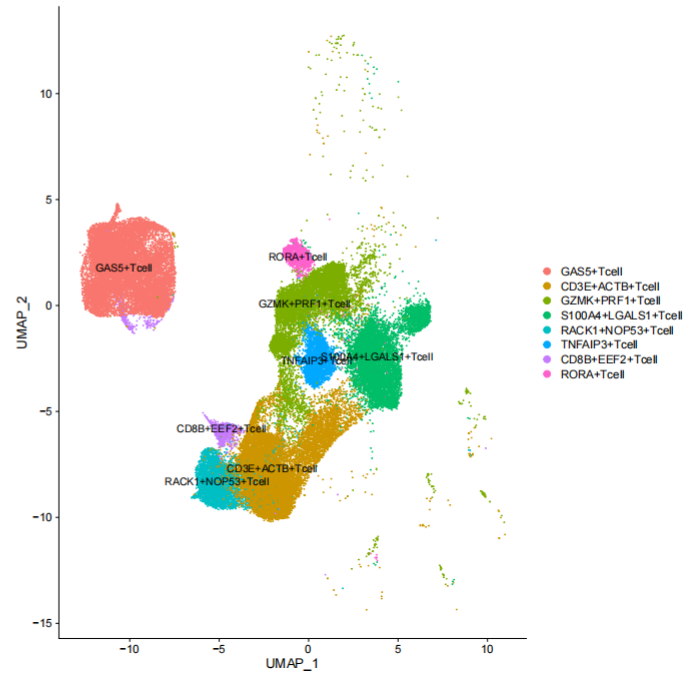

C

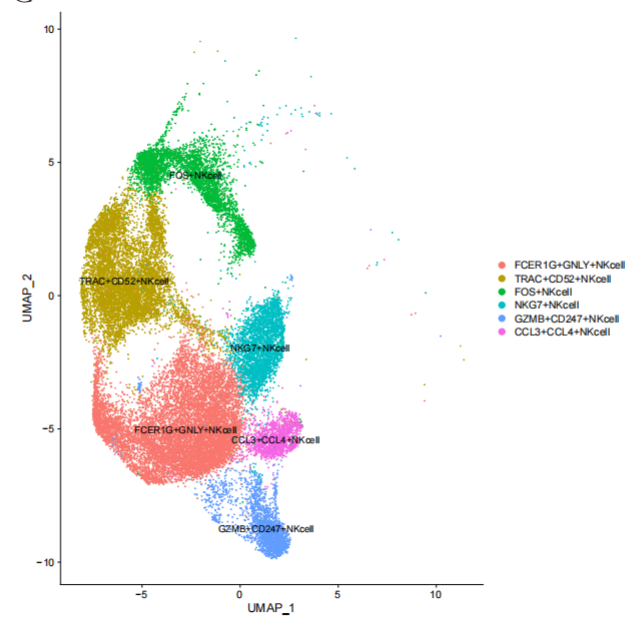

Supplement: Multimedia component 11 [file mmc11.pdf]

A

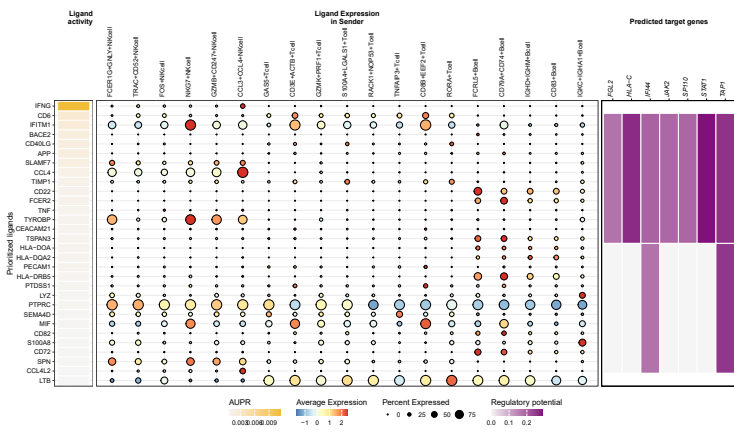

B

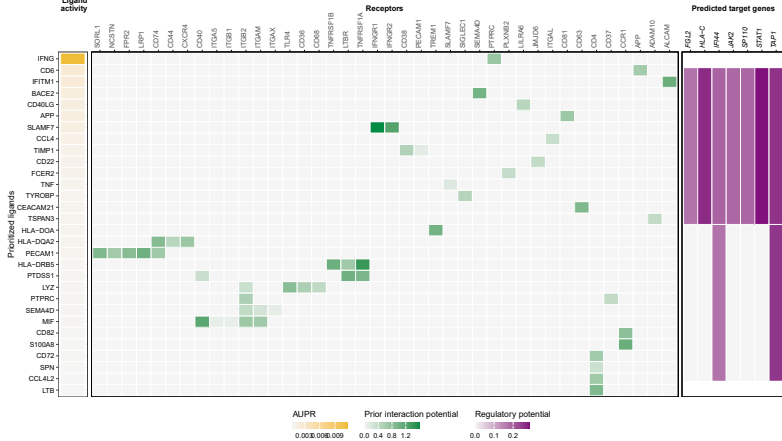

C

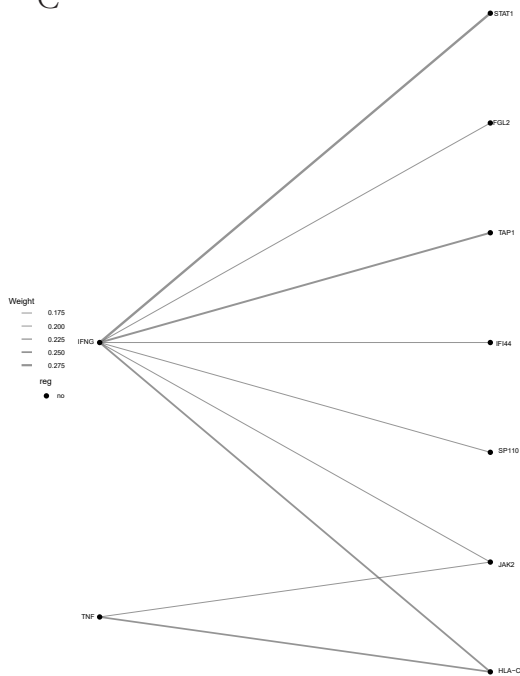

Supplement: Multimedia component 14 [file mmc14.pdf]

sJIA external set

sJIA internal set

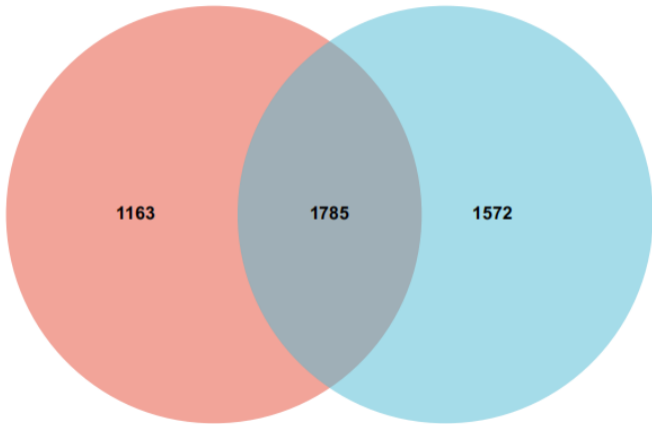

Supplement: Multimedia component 16 [file mmc16.pdf]

A

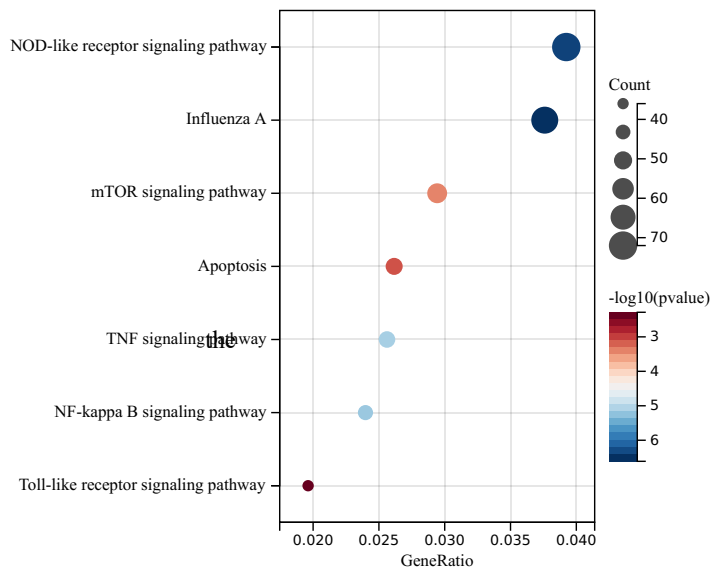

B

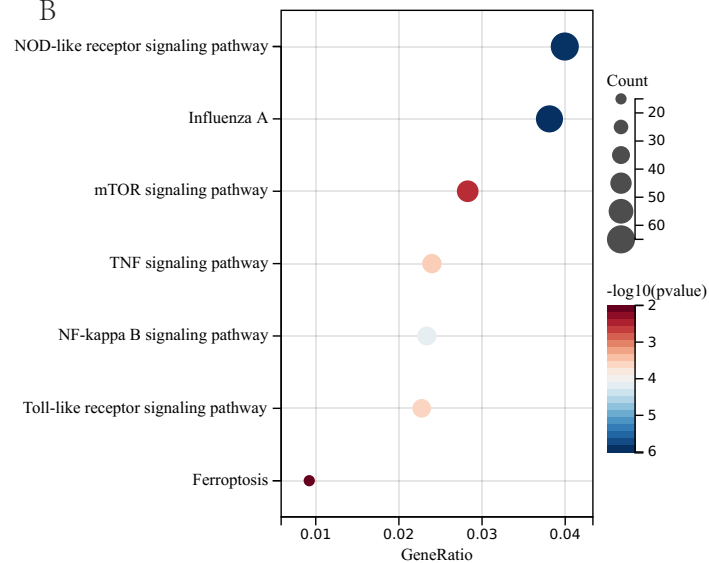

C

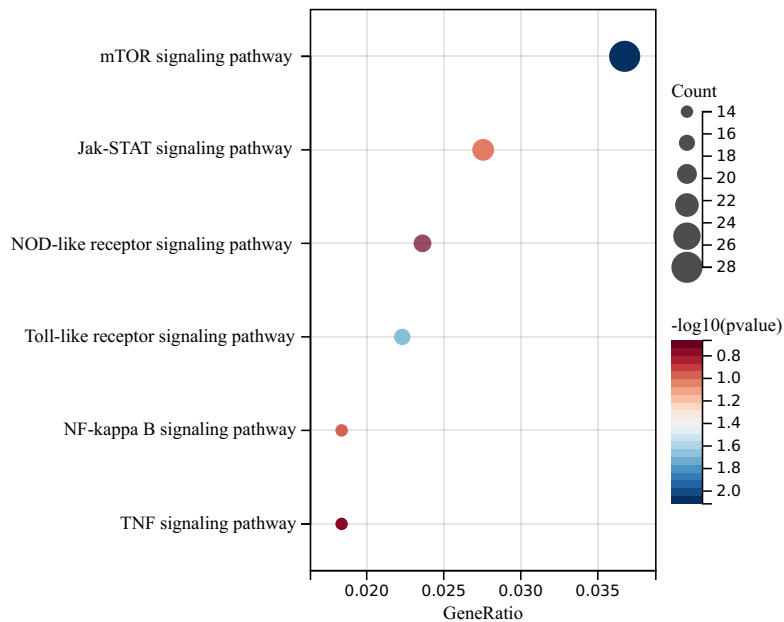

Supplement: Multimedia component 17 [file mmc17.pdf]

A

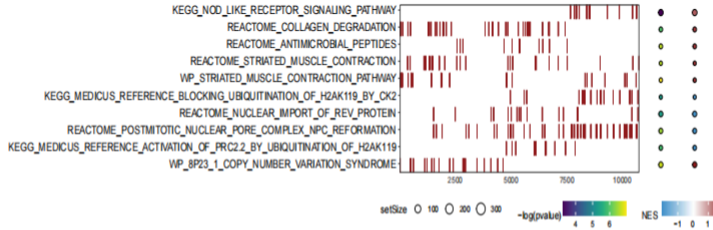

B

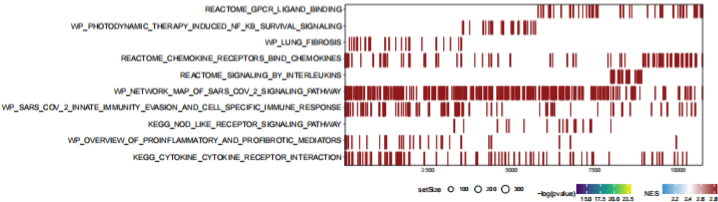

C

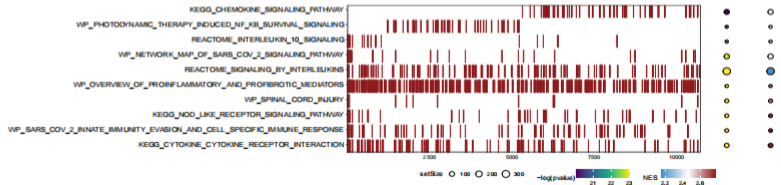

Supplement: Multimedia component 18 [file mmc18.pdf]

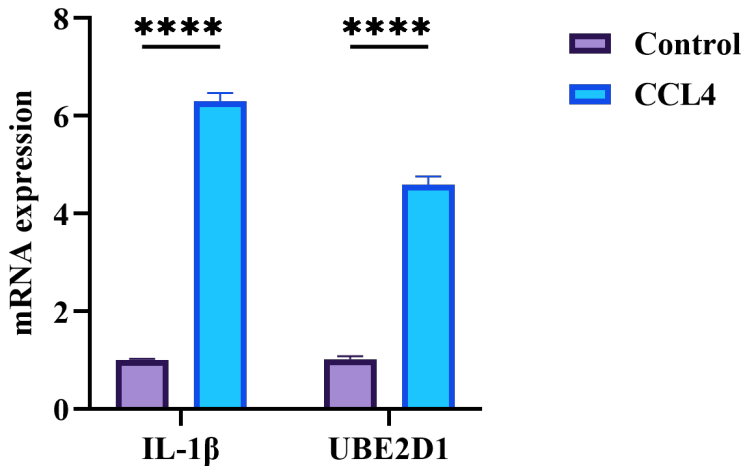

Supplement: Multimedia component 19 [file mmc19.pdf]

A

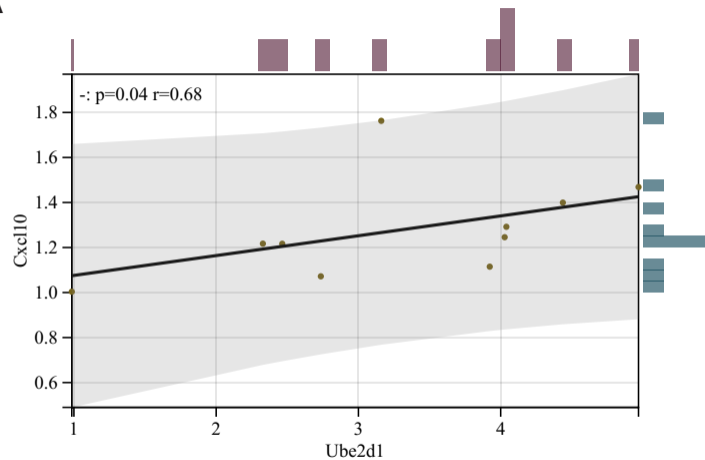

B

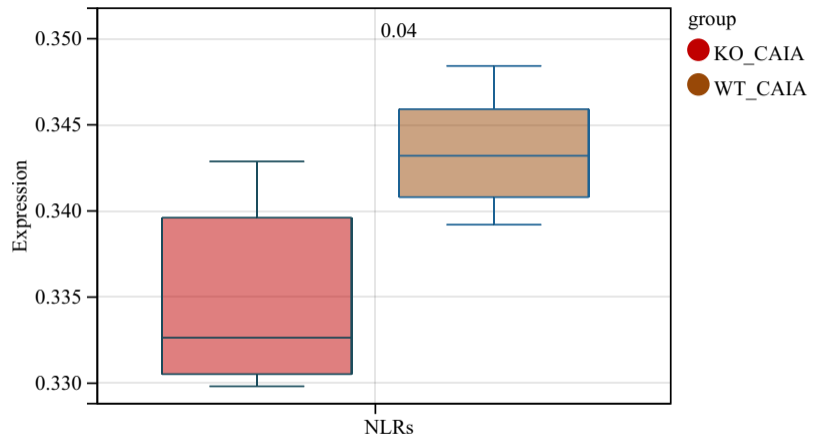

Supplement: Multimedia component 20 [file mmc20.pdf]

A

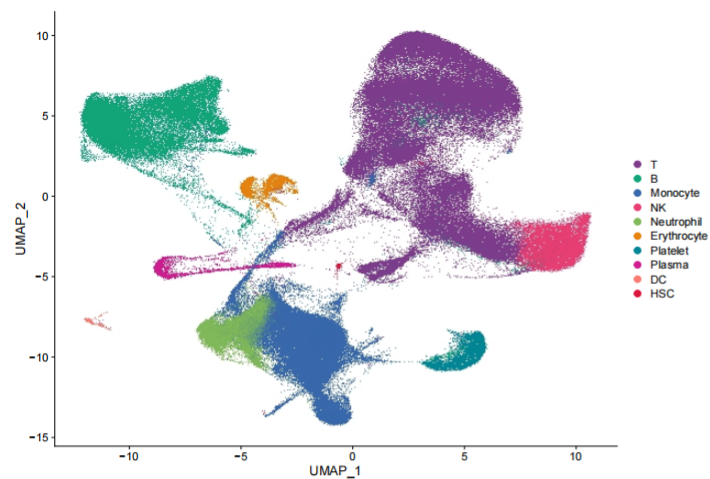

B

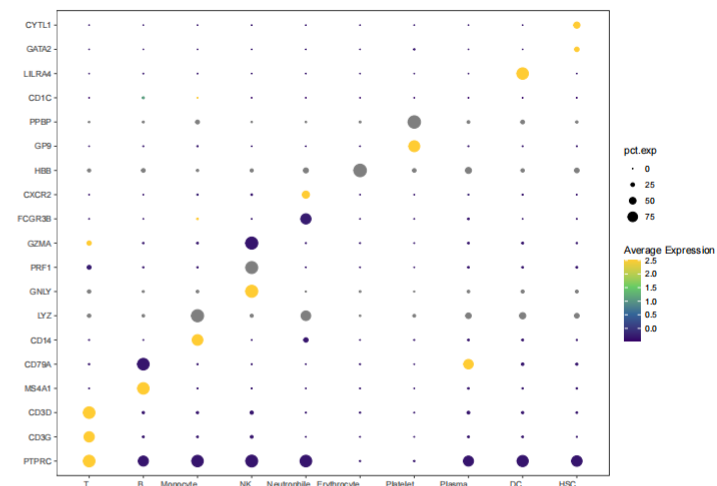

C

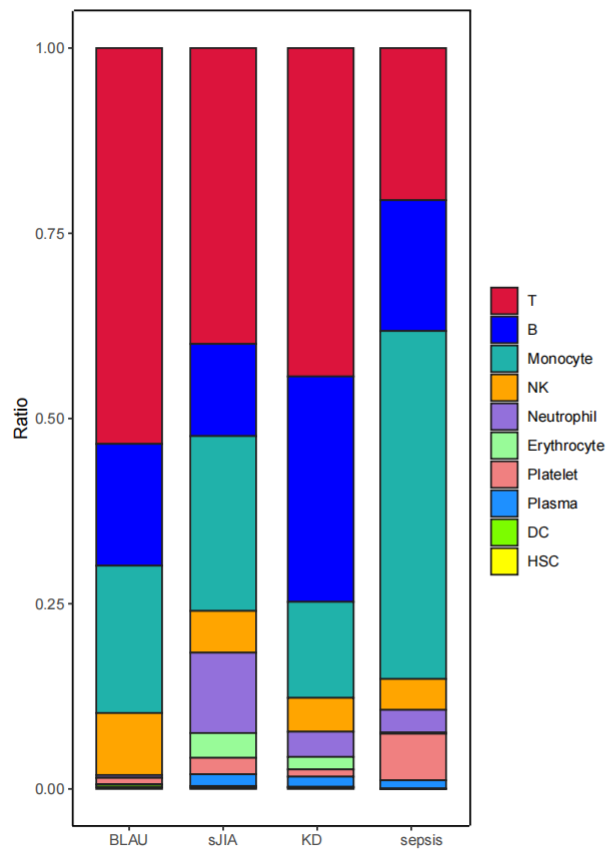

D

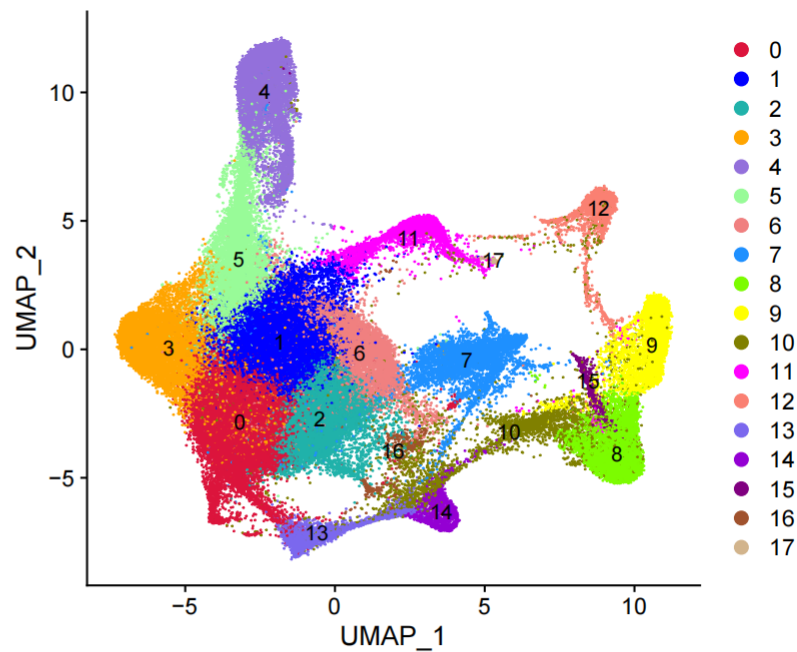

Supplement: Multimedia component 21 [file mmc21.pdf]

A

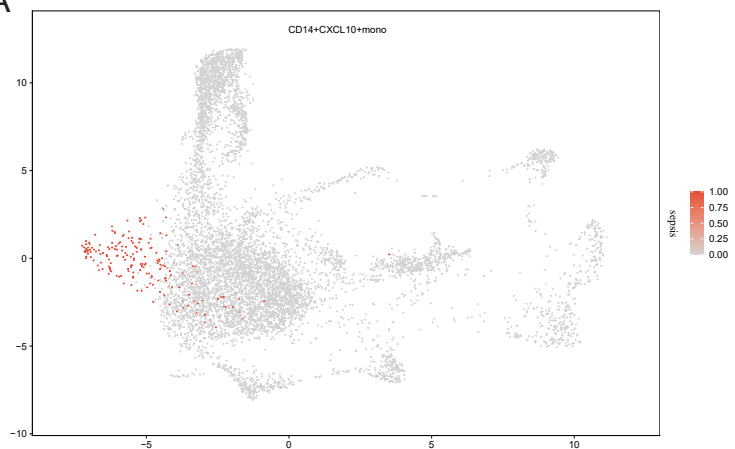

B

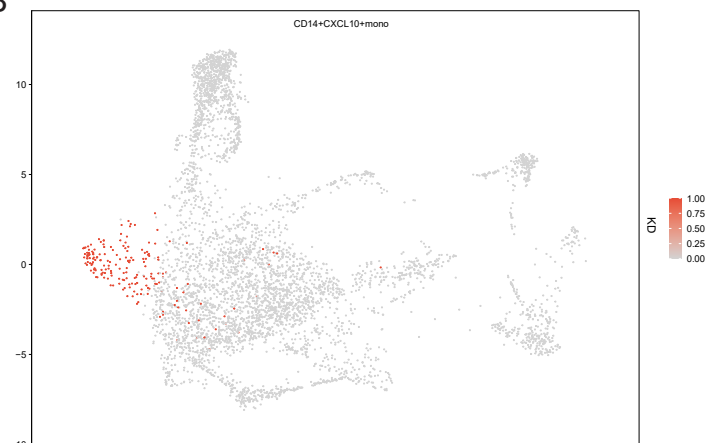

C

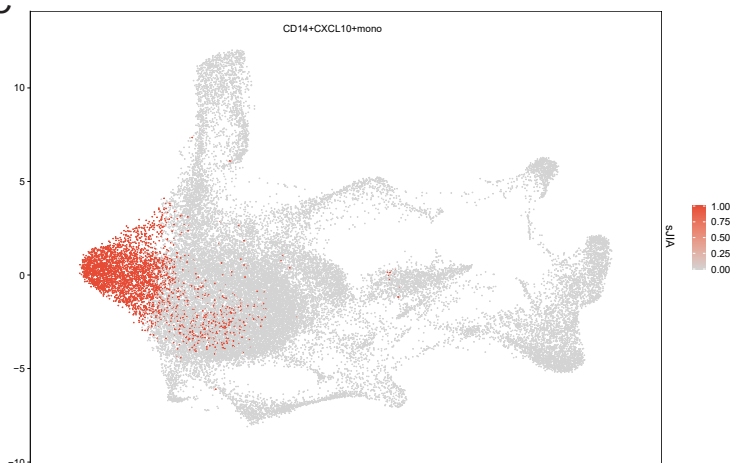

D

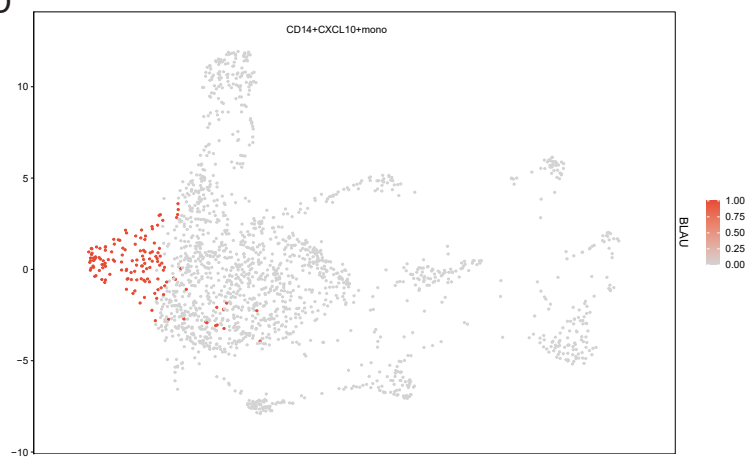

E

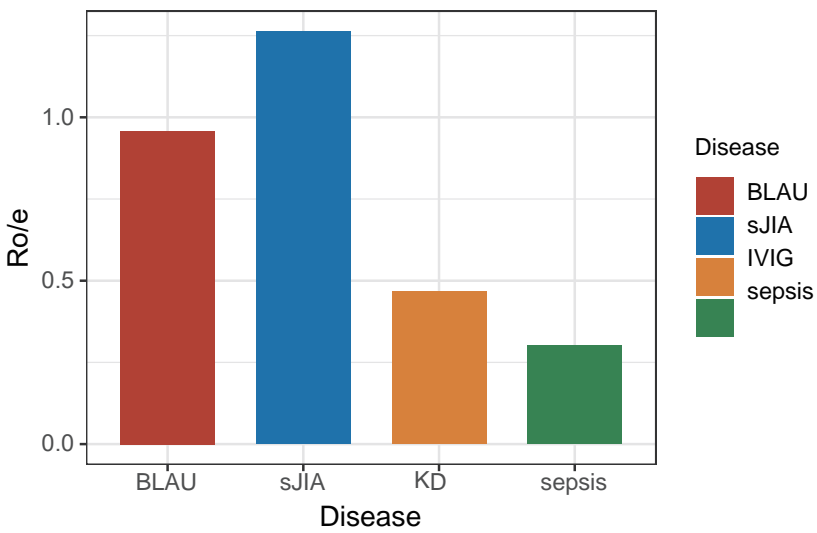

F

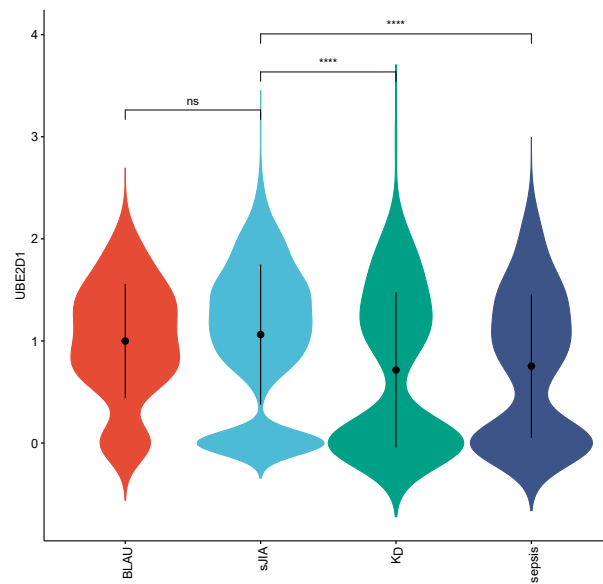

Supplement: Multimedia component 22 [file mmc22.pdf]
