# Supplementary material for: CD14+CXCL10+ monocytes are associated with peripheral immune network alterations in systemic juvenile idiopathic arthritis: From multiple centers
Source: Genes Dis. 2025 Nov 19;13(4):101942. doi: 10.1016/j.gendis.2025.101942 (PMC13091345; doi:10.1016/j.gendis.2025.101942)
Supplement: Multimedia component 9 [file mmc9.pdf]

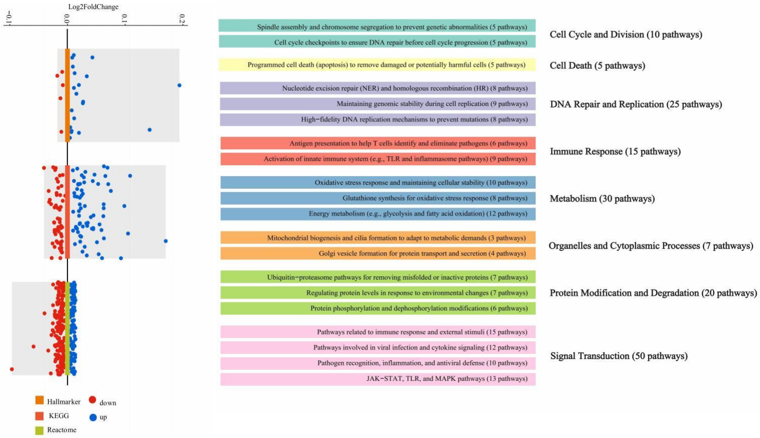

Log2FoldChange
-0.1
0.0
0.1
0.2
Hallmarker
KEGG
Reactome
down
up
Spindle assembly and chromosome segregation to prevent genetic abnormalities (5 pathways)
Cell cycle checkpoints to ensure DNA repair before cell cycle progression (5 pathways)
Cell Cycle and Division (10 pathways)
Programmed cell death (apoptosis) to remove damaged or potentially harmful cells (5 pathways)
Cell Death (5 pathways)
Nucleotide excision repair (NER) and homologous recombination (HR) (8 pathways)
Maintaining genomic stability during cell replication (9 pathways)
High−fidelity DNA replication mechanisms to prevent mutations (8 pathways)
DNA Repair and Replication (25 pathways)
Antigen presentation to help T cells identify and eliminate pathogens (6 pathways)
Activation of innate immune system (e.g., TLR and inflammasome pathways) (9 pathways)
Immune Response (15 pathways)
Oxidative stress response and maintaining cellular stability (10 pathways)
Glutathione synthesis for oxidative stress response (8 pathways)
Energy metabolism (e.g., glycolysis and fatty acid oxidation) (12 pathways)
Metabolism (30 pathways)
Mitochondrial biogenesis and cilia formation to adapt to metabolic demands (3 pathways)
Golgi vesicle formation for protein transport and secretion (4 pathways)
Organelles and Cytoplasmic Processes (7 pathways)
Ubiquitin−proteasome pathways for removing misfolded or inactive proteins (7 pathways)
Regulating protein levels in response to environmental changes (7 pathways)
Protein phosphorylation and dephosphorylation modifications (6 pathways)
Protein Modification and Degradation (20 pathways)
Pathways related to immune response and external stimuli (15 pathways)
Pathways involved in viral infection and cytokine signaling (12 pathways)
Pathogen recognition, inflammation, and antiviral defense (10 pathways)
JAK−STAT, TLR, and MAPK pathways (13 pathways)
Signal Transduction (50 pathways)
