# Supplementary material for: CD14+CXCL10+ monocytes are associated with peripheral immune network alterations in systemic juvenile idiopathic arthritis: From multiple centers
Source: Genes Dis. 2025 Nov 19;13(4):101942. doi: 10.1016/j.gendis.2025.101942 (PMC13091345; doi:10.1016/j.gendis.2025.101942)
Supplement: Multimedia component 12 [file mmc12.pdf]

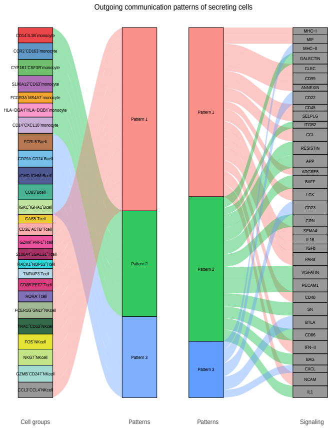
Outgoing communication patterns of secreting cells
CD14^IL1B^monocyte
CCR2^CD163^monocyte
CYP1B1^CSF3R^monocyte
S100A12^CD63^monocyte
FCGR3A^MS4A7^monocyte
HLA−DQA1^HLA−DQB1^monocyte
CD14^CXCL10^monocyte
FCRL5^Bcell
CD79A^CD74^Bcell
IGHD^IGHM^Bcell
CD83^Bcell
IGKC^IGHA1^Bcell
GAS5^Tcell
CD3E^ACTB^Tcell
GZMK^PRF1^Tcell
S100A4^LGALS1^Tcell
RACK1^NOP53^Tcell
TNFAIP3^Tcell
CD8B^EEF2^Tcell
RORA^Tcell
FCER1G^GNLY^NKcell
TRAC^CD52^NKcell
FOS^NKcell
NKG7^NKcell
GZMB^CD247^NKcell
CCL3^CCL4^NKcell
Pattern 1
Pattern 2
Pattern 3
Pattern 1
Pattern 2
Pattern 3
MHC−I
MIF
MHC−II
GALECTIN
CLEC
CD99
ANNEXIN
CD22
CD45
SELPLG
ITGB2
CCL
RESISTIN
APP
ADGRE5
BAFF
LCK
CD23
GRN
SEMA4
IL16
TGFb
PARs
VISFATIN
PECAM1
CD40
SN
BTLA
CD86
IFN−II
BAG
CXCL
NCAM
IL1
Cell groups
Patterns
Patterns
Signaling
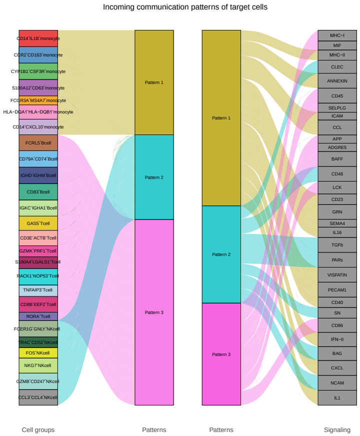
Incoming communication patterns of target cells
CD14^IL1B^monocyte
CCR2^CD163^monocyte
CYP1B1^CSF3R^monocyte
S100A12^CD63^monocyte
FCGR3A^MS4A7^monocyte
HLA−DQA1^HLA−DQB1^monocyte
CD14^CXCL10^monocyte
FCRL5^Bcell
CD79A^CD74^Bcell
IGHD^IGHM^Bcell
CD83^Bcell
IGKC^IGHA1^Bcell
GAS5^Tcell
CD3E^ACTB^Tcell
GZMK^PRF1^Tcell
S100A4^LGALS1^Tcell
RACK1^NOP53^Tcell
TNFAIP3^Tcell
CD8B^EEF2^Tcell
RORA^Tcell
FCER1G^GNLY^NKcell
TRAC^CD52^NKcell
FOS^NKcell
NKG7^NKcell
GZMB^CD247^NKcell
CCL3^CCL4^NKcell
Pattern 1
Pattern 2
Pattern 3
Pattern 1
Pattern 2
Pattern 3
MHC−I
MIF
MHC−II
CLEC
ANNEXIN
CD45
SELPLG
ICAM
CCL
APP
ADGRE5
BAFF
CD48
LCK
CD23
GRN
SEMA4
IL16
TGFb
PARs
VISFATIN
PECAM1
CD40
SN
CD86
IFN−II
BAG
CXCL
NCAM
IL1
Cell groups
Patterns
Patterns
Signaling
