# Supplementary material for: CD14+CXCL10+ monocytes are associated with peripheral immune network alterations in systemic juvenile idiopathic arthritis: From multiple centers
Source: Genes Dis. 2025 Nov 19;13(4):101942. doi: 10.1016/j.gendis.2025.101942 (PMC13091345; doi:10.1016/j.gendis.2025.101942)
Supplement: Multimedia component 15 [file mmc15.pdf]

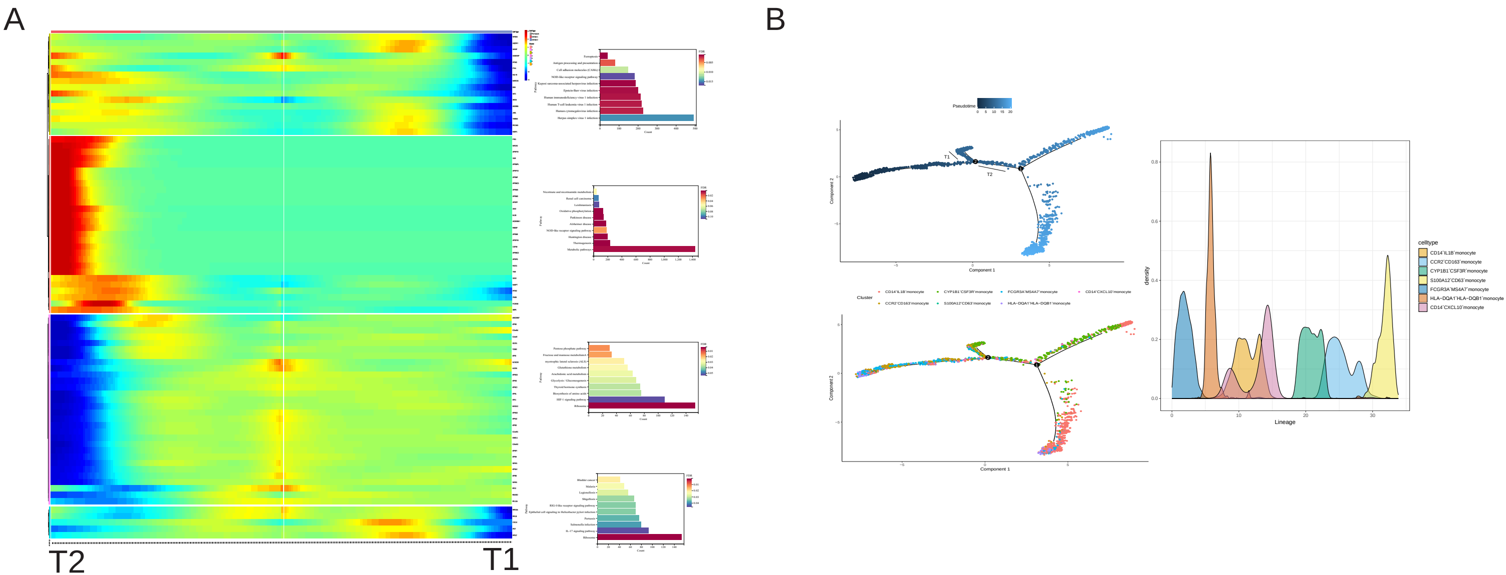
A
B
T2
T1
Ferroptosis
Antigen processing and presentation
Cell adhesion molecules (CAMs)
NOD-like receptor signaling pathway
Kaposi sarcoma-associated herpesvirus infection
Epstein-Barr virus infection
Human immunodeficiency virus 1 infection
Human T-cell leukemia virus 1 infection
Human cytomegalovirus infection
Herpes simplex virus 1 infection
Pathway
Count
FDR
Nicotinate and nicotinamide metabolism
Renal cell carcinoma
Leishmaniasis
Oxidative phosphorylation
Parkinson disease
Alzheimer disease
NOD-like receptor signaling pathway
Huntington disease
Thermogenesis
Metabolic pathways
Pentose phosphate pathway
Fructose and mannose metabolism
Amyotrophic lateral sclerosis (ALS)
Glutathione metabolism
Arachidonic acid metabolism
Glycolysis / Gluconeogenesis
Thyroid hormone synthesis
Biosynthesis of amino acids
HIF-1 signaling pathway
Ribosome
Bladder cancer
Malaria
Legionellosis
Shigellosis
RIG-I-like receptor signaling pathway
Epithelial cell signaling in Helicobacter pylori infection
Pertussis
Salmonella infection
IL-17 signaling pathway
Ribosome
Pseudotime
T1
T2
Component 1
Component 2
Cluster
CD14+IL1B+monocyte
CCR2+CD163+monocyte
CYP1B1+CSF3R+monocyte
S100A12+CD63+monocyte
FCGR3A+MS4A7+monocyte
HLA−DQA1+HLA−DQB1+monocyte
CD14+CXCL10+monocyte
density
Lineage
celltype
