# Supplementary material for: CD14+CXCL10+ monocytes are associated with peripheral immune network alterations in systemic juvenile idiopathic arthritis: From multiple centers
Source: Genes Dis. 2025 Nov 19;13(4):101942. doi: 10.1016/j.gendis.2025.101942 (PMC13091345; doi:10.1016/j.gendis.2025.101942)
Supplement: Multimedia component 13 [file mmc13.pdf]

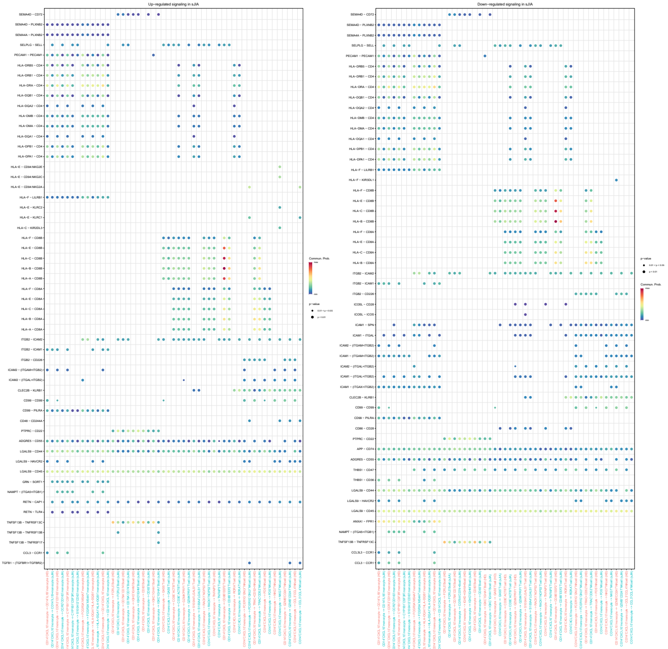

Up−regulated signaling in sJIA
SEMA4D − CD72
SEMA4D − PLXNB2
SEMA4A − PLXNB2
SELPLG − SELL
PECAM1 − PECAM1
HLA−DRB5 − CD4
HLA−DRB1 − CD4
HLA−DRA − CD4
HLA−DQB1 − CD4
HLA−DQA2 − CD4
HLA−DMB − CD4
HLA−DMA − CD4
HLA−DQA1 − CD4
HLA−DPB1 − CD4
HLA−DPA1 − CD4
HLA−E − CD94:NKG2E
HLA−E − CD94:NKG2C
HLA−E − CD94:NKG2A
HLA−F − LILRB1
HLA−E − KLRC2
HLA−E − KLRC1
HLA−C − KIR2DL3
HLA−F − CD8B
HLA−E − CD8B
HLA−C − CD8B
HLA−B − CD8B
HLA−A − CD8B
HLA−F − CD8A
HLA−E − CD8A
HLA−C − CD8A
HLA−B − CD8A
HLA−A − CD8A
ITGB2 − ICAM2
ITGB2 − ICAM1
ITGB2 − CD226
ICAM2 − (ITGAM+ITGB2)
ICAM2 − (ITGAL+ITGB2)
CLEC2B − KLRB1
CD99 − CD99
CD99 − PILRA
CD48 − CD244A
PTPRC − CD22
ADGRE5 − CD55
LGALS9 − CD44
LGALS9 − HAVCR2
LGALS9 − CD45
GRN − SORT1
NAMPT − (ITGA5+ITGB1)
RETN − CAP1
RETN − TLR4
TNFSF13B − TNFRSF13C
TNFSF13B − TNFRSF13B
TNFSF13B − TNFRSF17
CCL3 − CCR1
TGFB1 − (TGFBR1+TGFBR2)
Commun. Prob.
max
min
p−value
0.01 < p < 0.05
p < 0.01
Down−regulated signaling in sJIA
SEMA4D − CD72
SEMA4D − PLXNB2
SEMA4A − PLXNB2
SELPLG − SELL
PECAM1 − PECAM1
HLA−DRB5 − CD4
HLA−DRB1 − CD4
HLA−DRA − CD4
HLA−DQB1 − CD4
HLA−DQA2 − CD4
HLA−DMB − CD4
HLA−DMA − CD4
HLA−DQA1 − CD4
HLA−DPB1 − CD4
HLA−DPA1 − CD4
HLA−F − LILRB1
HLA−F − KIR3DL1
HLA−F − CD8B
HLA−E − CD8B
HLA−C − CD8B
HLA−B − CD8B
HLA−F − CD8A
HLA−E − CD8A
HLA−C − CD8A
HLA−B − CD8A
ITGB2 − ICAM2
ITGB2 − ICAM1
ITGB2 − CD226
ICOSL − CD28
ICOSL − ICOS
ICAM1 − SPN
ICAM1 − ITGAL
ICAM2 − (ITGAM+ITGB2)
ICAM1 − (ITGAM+ITGB2)
ICAM2 − (ITGAL+ITGB2)
ICAM1 − (ITGAL+ITGB2)
ICAM1 − (ITGAX+ITGB2)
CLEC2B − KLRB1
CD99 − CD99
CD99 − PILRA
CD86 − CD28
PTPRC − CD22
APP − CD74
ADGRE5 − CD55
THBS1 − CD47
THBS1 − CD36
LGALS9 − CD44
LGALS9 − HAVCR2
LGALS9 − CD45
ANXA1 − FPR1
NAMPT − (ITGA5+ITGB1)
TNFSF13B − TNFRSF13C
CCL3L3 − CCR1
CCL3 − CCR1
p−value
0.01 < p < 0.05
p < 0.01
Commun. Prob.
max
min
